# Desumoylase SENP6 maintains osteochondroprogenitor homeostasis by suppressing the p53 pathway

**DOI:** 10.1038/s41467-017-02413-3

**Published:** 2018-01-10

**Authors:** Jianshuang Li, Di Lu, Hong Dou, Huadie Liu, Kevin Weaver, Wenjun Wang, Jiada Li, Edward T.H. Yeh, Bart O. Williams, Ling Zheng, Tao Yang

**Affiliations:** 1 Center for Cancer and Cell Biology, Van Andel Research Institute, Grand Rapids, MI 49503 USA; 2 Hubei Key Laboratory of Cell Homeostasis, College of Life Sciences, Wuhan University, Wuhan, 430072 Hubei P. R. China; 3 Center for Precision Medicine, Department of Medicine, University of Missouri, Columbia, MO 65212 USA; 4 State Key Laboratory of Medical Genetics and School of Life Sciences, Central South University, Changsha, 410078 Hunan P. R. China

## Abstract

The development, growth, and renewal of skeletal tissues rely on the function of osteochondroprogenitors (OCPs). Protein sumoylation/desumoylation has emerged as a pivotal mechanism for stem cell/progenitor homeostasis, and excessive sumoylation has been associated with cell senescence and tissue aging, but its role in regulating OCP function is unclear. Here we show that postnatal loss of the desumoylase SUMO1/sentrin-specific peptidase 6 (SENP6) causes premature aging. OCP-specific SENP6 knockout mice exhibit smaller skeletons, with elevated apoptosis and cell senescence in OCPs and chondrocytes. In *Senp6*^*‒/‒*^ cells, the two most significantly elevated pathways are p53 signaling and senescence-associated secreted phenotypes (SASP), and *Trp53* loss partially rescues the skeletal and cellular phenotypes caused by *Senp6* loss. Furthermore, SENP6 interacts with, desumoylates, and stabilizes TRIM28, suppressing p53 activity. Our data reveals a crucial role of the SENP6–p53 axis in maintaining OCP homeostasis during skeletal development.

## Abstract (web summary)

Osteochondroprogenitors are essential cells for skeletal development and homeostasis. Here the authors show that the desumoylase SENP6 suppresses p53 activity by desumoylating and stabilising TRIM28, and that SENP6 ablation leads to skeletal abnormalities, senescence in osteochondroprogenitors and chondrocytes, and premature ageing.

## Introduction

Osteochondroprogenitor cells (OCPs) are derived from the mesenchymal cell lineage and give rise to chondrocytes or osteoblasts. The renewal and maintenance of OCPs are crucial for embryonic skeletal development as well as for postnatal skeletal growth and homeostasis^1,2^. Defective OCP maintenance is considered an important cause of skeletal aging, as the exhaustion of progenitors or stem cells is one of the ultimate culprits of tissue/organism aging^3^.

Emerging evidence indicates that the components of the sumoylation/desumoylation machineries play important roles in stem cell and progenitor maintenance and in tissue aging. For example, the sumoylation E2 enzyme UBC9 is essential for maintaining the survival and pluripotency of embryonic stem cells (ESCs)^4^. SENP2, a SUMO-deconjugating enzyme (desumoylase), sustains the quiescent status of muscle satellite cells and regulates placental trophoblast progenitor expansion and differentiation^5,6^. Overexpression of SUMO2/3 or PIASγ (SUMO E3 ligases) or suppression of the SENP1, 2, or 7 can promote cell senescence^7–9^. In addition, global increases in sumoylation have been detected in tissues derived from aged rats^10^.

The role of the sumoylation/desumoylation processes in bone and cartilage homeostasis has been suggested by several recent studies. UBC9 inhibits bone morphogenetic protein (BMP)-induced osteoblast differentiation in cultures partly by enhancing SMAD4 sumoylation^11^, and SENP3 promotes osteogenesis by desumoylating RbBP5 to activate the expression of *HOX* genes^12^. Moreover, a genome­wide association study (GWAS) has shown that a single nucleotide polymorphism (SNP) in the upstream sequence of the *SENP6* locus is one of the most significant genetic markers associated with severe osteoarthritis^13^, a disease highly associated with aging. Also, significantly lower SENP6 expression has been found in human osteoarthritic joints^14^.

SENP6 is localized in the nucleoplasm and has a preference for SUMO2/3-modified substrates; it regulates genome stability, cell division, and autoimmune responses, and is involved in adult hematopoietic stem cell renewal^15–19^. In this study, we report that SENP6 is a crucial regulator of the aging process and OCP maintenance during skeletal development. The skeletal phenotype caused by *Senp6* loss can be partially rescued by p53 loss. We further found that SENP6 suppresses p53 activity through interacting with, desumoylating, and stabilizing TRIM28 (tripartite motif-containing 28; also called KAP1 or TIF1β).

## Results

### Loss of *Senp6* in adult mice leads to premature aging

**Fig. 1:**
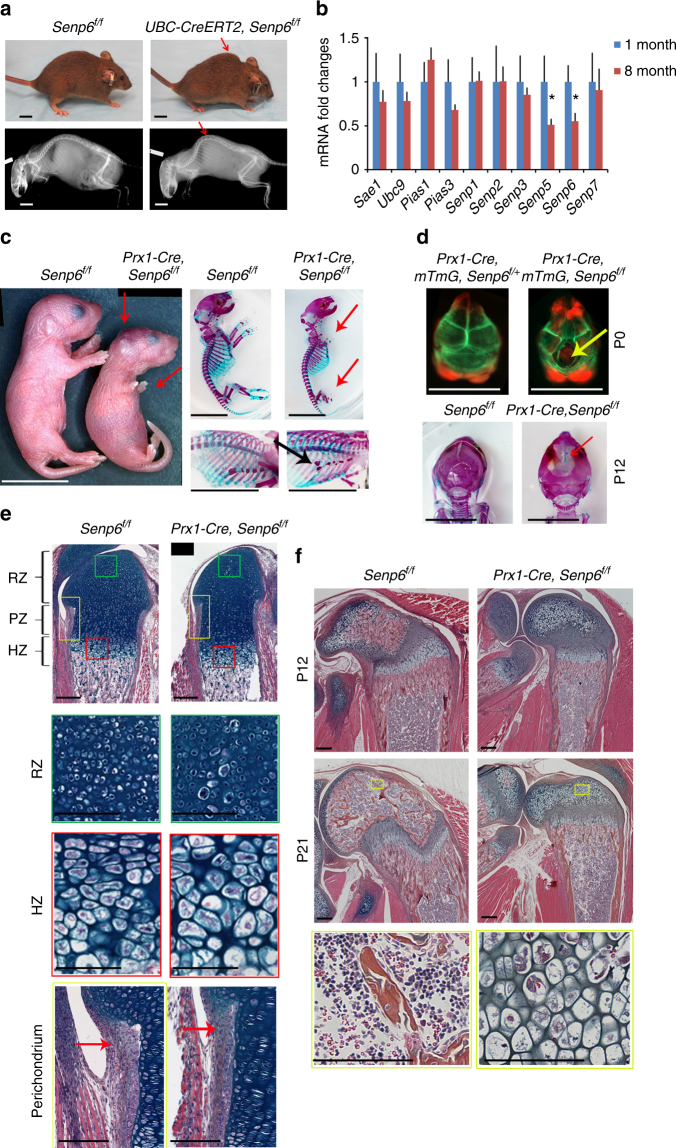
*Senp6* loss caused premature aging and skeletal developmental defects. **a** Four-momth-old *UBC-CreERT2;Senp6*^*f/f*^ mice developed kyphosis (red arrows) after tamoxifen-induced gene deletion (scale bars = 1 cm). **b** Expression of sumoylation system component genes in BMSCs derived from young (1 month) and older (8 months) mice (*n* = 4, error bars = standard deviation, *p < 0.05, Student’s *t*-test). **c** P0 *Prx1-Cre;Senp6*^*f/f*^ mice show smaller limbs (red arrows), asymmetric patterning, and decreased ossification of the sternum (black arrow). Scale bars = 1 cm. **d** Undermineralized cranial bone in P0 *Prx1-Cre;Senp6*^*f/f*^*;mTmG* mice (upper panel). The green fluorescence indicates the region with active Cre expression; the yellow arrow denotes the opening of the skull. In P12 *Prx1-Cre;Senp6*^*f/f*^ mice (lower panel), the red arrow indicates the Alizarin red–negative region of the skull. Scale bar = 1 cm. **e** A distal femur of a P0 *Prx1-Cre;Senp6*^*f/f*^ mouse shows a reduced growth plate diameter and enlarged chondrocytes in the resting zone (RZ) and hypertrophic zone (HZ), as well as a thinner perichondrium (red arrows). The colored boxes are enlarged and shown in the lower panels; scale bars = 100 μm for the growth plate and 50 μm for the rest. **f** *Prx1-Cre;Senp6*^*f/f*^ mice showed dramatically delayed formation of the secondary ossification center (SOC) at P12 and P21. The yellow boxes are enlarged and shown in the lower panels. Scale bars = 100 μm for the growth plate and 50 μm for enlarged yellow boxes

Global *Senp6* loss in mice causes early embryonic death (data not shown). In order to assess in which postnatal tissues/organs SENP6 has a crucial role, we first bred *Senp6* floxed mice (*Senp6*^*f/f*^) with *UBC-CreERT2* mice (a tamoxifen-induced gene deleter active in all tissues) (Supplementary Figure 1)^20^. Upon tamoxifen injection at 2 months of age, the *UBC-CreERT2*;*Senp6*^*f/f*^ mice progressively developed premature aging complexions, including skin laxity and spotty pigmentation, as well as severe kyphosis at 4 months (Fig. 1a). The kyphosis phenotype implied that SENP6 may play a role in postnatal skeletal maintenance, where mesenchymal stem cells/progenitors have an important contribution^21^. Further, we detected a significant age-related decrease of *Senp6* expression in bone marrow stromal cells (BMSCs), a cell population rich in mesenchymal stem cell/progenitors, suggesting that SENP6 levels may be associated with the homeostasis of mesenchymal stem cells/progenitors during aging (Fig. 1b).

### Loss of *Senp6* in OCPs impairs embryonic skeletal growth

Because stem cell/progenitor exhaustion is one of ultimate hallmarks of aging^3^, and because the sumoylation/desumoylation pathway has been associated with stem cell maintenance and differentiation^4–6^, the aging phenotypes caused by SENP6 loss intrigued us to further assess the specific functions of *Senp6* in mesenchymal stem cells/progenitors. We first generated *Dermo1-Cre;Senp6*^*f/f*^ mice, which had a distinctively small body size and complete penetrance of perinatal death (Supplementary Figure 2). To circumvent the embryonic death, we generated *Prx1-Cre;Senp6*^*f/f*^ mice, in which the *Senp6* gene is specifically deleted in the OCPs of the limb, midline, and a portion of cranial bones, but most of the axial skeleton is not affected^22^. *Prx1-Cre;Senp6*^*f/f*^ mice were born at the expected Mendelian ratio. The effectiveness of Cre-mediated gene deletion in these mice was confirmed by qRT-PCR in the limb and calvarial tissues, as well as by the GFP expression from the *mTmG* allele bred with this line (*Prx1-Cre;Senp6*^*f/f*^*;mTmG*) (Supplementary Figure 3)^23^.

*Prx1-Cre;Senp6*^*f/f*^ mice had severe limb developmental defects at birth, and about 80% died before weaning, likely due to the difficulty in feeding as a result of their limb defects. Protruding blood sacks on the skulls were noted in the P0 mutant mice, suggesting defective cranial bone development (Fig. 1c). Severe shortening of forelimbs and hindlimbs, abnormally patterned sternums, and distinctively undermineralized skulls were noted in the *Prx1-Cre;Senp6*^*f/f*^ mice at P0 and P12 (Fig. 1c, d**)**. We also found that P12 *Prx1-Cre;Senp6*^*f/f*^ mice had an undermineralized epiphysis (indicated by the lack of alizarin red staining, Supplementary Figure 4), suggesting that these mice had delayed formation of the secondary ossification center (SOC).

Histological staining showed that in the growth plate of P0 *Prx1-Cre;Senp6*^*f/f*^ mice, the lengths of the resting zone (RZ), proliferating zone (PZ), and hypertrophic zone (HZ) were only slightly changed, but the width of the growth plate was dramatically reduced relative to littermate controls (Fig. 1e, Supplementary Figure 5a, b). Moreover, we observed enlarged chondrocytes in the RZ and HZ of the *Prx1-Cre;Senp6*^*f/f*^ growth plate (Fig. 1e). The thickness of the perichondrium, which is the OCP-rich tissue surrounding the growth plate and provides new chondrocytes to support growth plate radial expansion, was also markedly reduced (Fig. 1e, Supplementary Figure 5c). The E18.5 *Dermo1-Cre;Senp6*^*f/f*^ mice showed growth plate and chondrocyte phenotypes similar to those of *Prx1-Cre;Senp6*^*f/f*^ mice (Supplementary Figure 6a, b).

*Prx1-Cre;Senp6*^*f/f*^ mice showed a severely delayed formation of the SOC, a bony structure developed from the center of the epiphysis separating the articular cartilage from the growth plate^1^. At P12, while the SOCs were clearly established in the proximal femurs of control (*Senp6*^*f/f*^) mice, the epiphysis of the *Prx1-Cre;Senp6*^*f/f*^ mice showed no sign of ossification (Fig. 1f). At P21, when SOC formation was finished in control mice, this process was just initiated in the mutant mice (Fig. 1f). The onset of SOC formation requires the development of cartilage canals, which are invaginated perichondrium invading into the epiphysis center, and they bring OCPs, vessels, and hematopoietic cells for osteogenesis and the establishment of bone marrow^24^. The impaired perichondrium expansion and lack of cartilage canals may contribute to the delay in SOC formation in *Prx1-Cre;Senp6*^*f/f*^ mice.

### *Senp6* loss leads to defective cell homeostasis and DNA repair

**Fig. 2:**
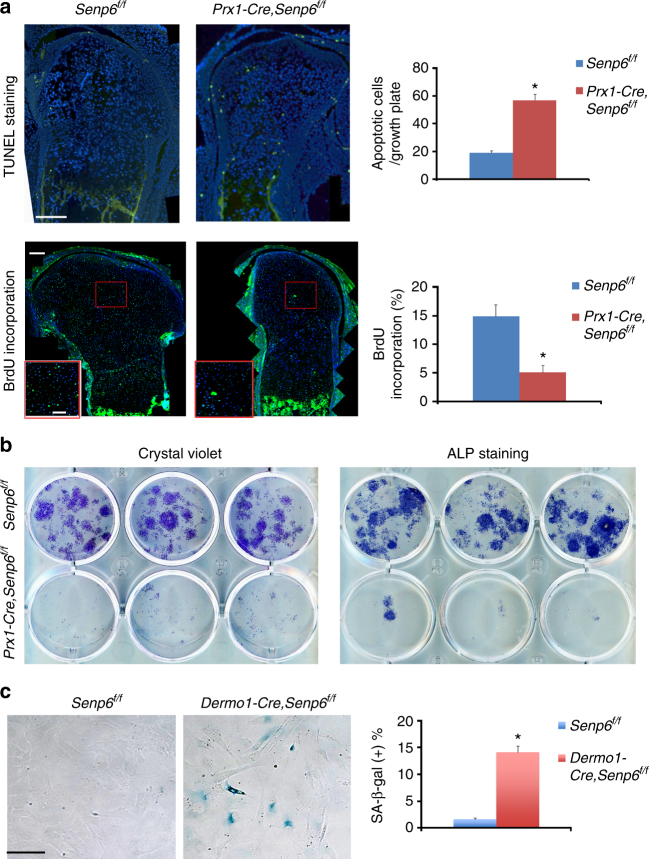
Dysregulated OCP and chondrocyte homeostasis caused by *Senp6* loss. **a** The growth plate of P0 *Prx1-Cre;Senp6*^*f/f*^ mice showed an enhanced apoptosis (upper panel) and decreased proliferation (lower panel). The red boxed regions were enlarged and are shown in the lower-right corner of each image. Scale bars = 100 μm for growth plate and 50 μm for the enlarged red boxes. Representative images are on the left and quantifications on the right (*n* = 3, error bars = standard deviation, *p < 0.05, Student’s *t*-test). **b** *Prx1-Cre;Senp6*^*f/f*^ bone marrow had a decreased number of CFU-F and CFU-O (*n* = 3). **c** Rib chondrocytes cultured from *Dermo1-Cre;Senp6*^*f/f*^ mouse showed an augmented cell senescence (SA-β-gal staining); representative images are at the left and quantification at the right. Scale bar = 50 μm; *n* = 3, error bars = standard deviation; *p < 0.05 relative to *Senp6*^*f/f*^ (control), Student’s *t*-test

Compared with control mice, the growth plate of P0 *Prx1-Cre;Senp6*^*f/f*^ mice showed a significantly more TUNEL-positive apoptotic cells, especially in the RZ and periarticular area (Fig. 2a), as well as a significantly fewer BrdU-positive proliferating cells, most notably in the PZ (Fig. 2a). Consistently, total bone marrow from 1-month-old *Prx-1-Cre;Senp6*^*f/f*^ mice showed dramatically fewer colony forming unit-fibroblast (CFU-F; adhered stromal cells rich in OCPs) and CFU-osteoblast (CFU-O) relative to control (*Senp6*^*f/f*^) mice (Fig. 2b). Similar results were confirmed in the total bone marrow cultures using adenovirus-Cre (Ad-Cre)-mediated *Senp6* deletion (Supplementary Figure 7a). Moreover, the *Senp6*-deleted BMSCs showed a flatter growth curve that reached a plateau earlier than the BMSCs from WT controls (Supplementary Figure 7b). These data collectively suggest that *Senp6* loss affected both OCP expansion and OCP proportion in the bone marrow.

**Fig. 3:**
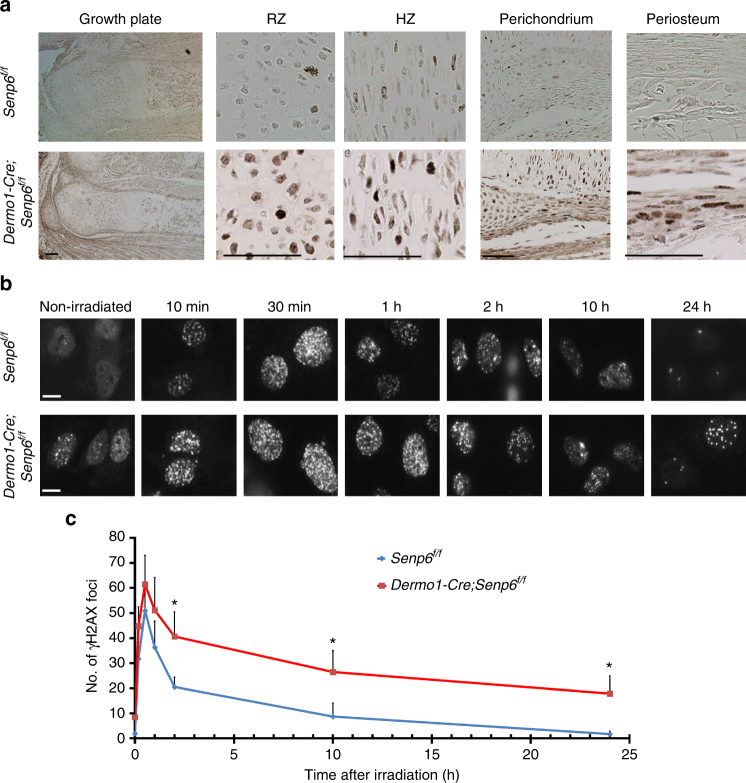
*Senp6* loss delayed DNA damage repair. **a** *Dermo1-Cre;Senp6*^*f/f*^ mice (E18.5) had more γH2AX-positive cells in the growth plate RZ, PZ, perichondrium, and periosteum than did *Senp6*^*f/f*^ controls; scale bars = 100 μm for the growth plate (left) and 50 μm for the rest. **b** *Dermo1-Cre;Senp6*^*f/f*^ mice showed an increased basal level of γH2AX foci (non-irradiated), higher sensitivity to irradiation (at 10 min), and lower clearance of γH2AX foci during recovery at 30 min to 24 h (*n* = 3; 50 areas of each slide were quantified, error bars = standard deviation, *p < 0.05, relative to control, Student’s *t*-test); scale bars = 10 μm

The primary rib chondrocyte of early passage (passage 0) from *Dermo1-Cre;Senp6*^*f/f*^ mice had significantly more senescence-associated β-galactosidase (SA-β-gal)-positive cells than the controls did (Fig. 2c). Increased SA-β-gal-positive cells were also detected in the growth plate and bone marrow sections of E18.5 *Dermo1-Cre;Senp6*^*f/f*^ mice (Supplementary Figure 8). This is consistent with the enlarged chondrocytes observed in the *Senp6-*deficient growth plate, because increased cell volume is one of the signs of cellular senescence. In addition, the expression of another senescence-related marker, γH2AX (also a DNA damage foci marker), was markedly increased in the mutant growth plates, especially in the RZ, PZ, perichondrium, and periosteum (Fig. 3a). Moreover, X-ray-irradiated *Dermo1-Cre;Senp6*^*f/f*^ chondrocytes showed dramatically more γH2AX foci per cell, and the clearance of γH2AX foci in the recovery phase was significantly delayed (Fig. 3b). It was previously reported that *SENP6* knockdown promotes RPA70 sumoylation, which leads to replication stress and DNA breaks^15^. In line with this, we found a marked increase of sumoylated RPA70 in the rib cartilage from E18.5 *Dermo1-Cre;Senp6*^*f/f*^ mice (Supplementary Figure 9). These data implied that the unresolved DNA damage, partly caused by RPA70 hypersumoylation, contributed to the elevated cell senescence and apoptosis of SENP6-deficient OCPs.

### *Senp6* loss activates the p53 and SASP pathways

**Fig. 4:**
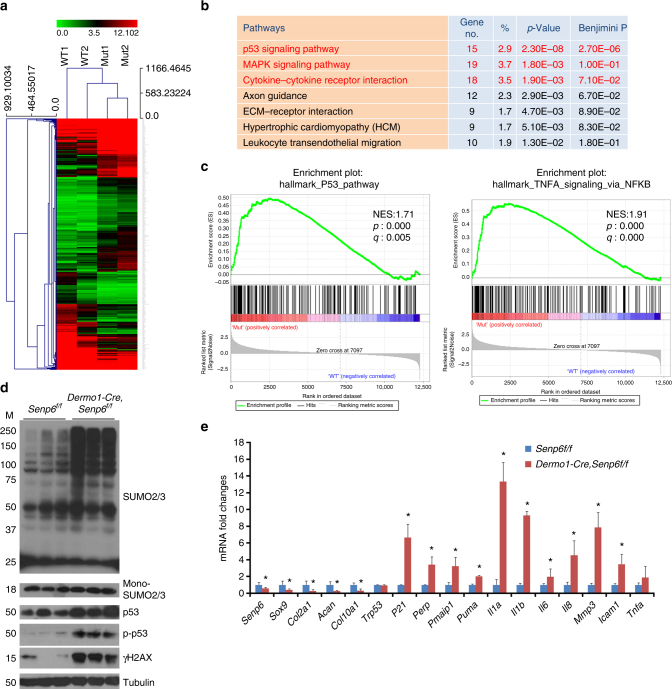
*Senp6* loss activated p53 signaling pathway and SASP. **a** Heatmap of RNA-seq gene expression profiles comparing *Dermo1-Cre;Senp6*^*f/f*^ and control primary rib chondrocytes (*n* = 2). **b** Up-regulated pathways based on DAVID GO analyses (*p*-values: Fisher Exact/EASE Score). **c** Enriched p53 pathway and TNFα-signaling-via-NFκB pathway in *Dermo1-Cre;Senp6*^*f/f*^ by GESA analysis (NES normalized enrichment score; *p*: nominal *p*-value; *q*: false discovery rate *q*-value). **d** *Dermo1-Cre;Senp6*^*f/f*^ rib cartilage (E18.5) showing higher total SUMO2/3-modified proteins, total and phosphorylated (S15) p53, and γH2AX relative to controls (*n* = 3). **e** Chondrocyte differentiation and proliferation markers, p53 downstream targets that promote apoptosis and senescence, and SASP markers were augmented in the rib cartilage of E18.5 *Dermo1-Cre;Senp6*^*f/f*^ mice (*n* = 3; error bars = standard deviation, *p < 0.05, relative to controls, Student’s *t*-test)

To understand the mechanisms underlying *Senp6*-loss-induced cell senescence and apoptosis, we compared the gene expression profiles of cultured rib chondrocytes from *Dermo1-Cre;Senp6*^*f/f*^ and *Senp6*^*f/f*^ (control) mice by RNA sequencing (RNA-seq). The DAVID Gene Ontology (GO) analysis revealed enriched gene clusters in the *Dermo1-Cre;Senp6*^*f/f*^ chondrocytes, including the p53 pathway, the MAPK pathway, and the cytokine–cytokine receptor interaction pathway (Fig. 4a, b). Similar results were obtained from the Gene Set Enrichment Analysis (GSEA) (Fig. 4c). In addition, the SUMO2/3-targeted proteins, total p53, phosphorylated p53 (Ser15), and γH2AX protein were significantly higher in *Dermo1-Cre;Senp6*^*f/f*^ samples than in those from control mice (Fig. 4d). However, total SUMO1-modified proteins were not distinctively altered in the *Dermo1-Cre;Senp6*^*f/f*^ cartilage (Supplementary Figure 10), in line with the fact that SENP6 has a strong preference for SUMO2/3-modified substrates^25^. Consistent with these data, the expression of p53 downstream targets that promote apoptosis and senescence (*P21*, *Perp*, *Pmaip1*, and *Puma*) and senescence-associated secreted phenotypes (SASP) markers (*Il1α*, *Il6*, *Il8*, *Mmp3*, and *Icam1*) were increased, while chondrocyte differentiation and proliferation markers (*Sox9*, *Col2a1*, *Acan*, and *Col10a1*) were decreased in mutant mice (Fig. 4e). However, in the rib cartilage, *Senp6* loss did not affect the expression of other key components of the sumoylation pathway, such as the sumoylation E1, E2, and E3 enzymes and the desumoylases (Supplementary Figure 11). In addition, compared with WT controls, the expression of *Cdkn2a*, which encodes the p16 protein (an important marker and regulator of cell senescence and aging), had a 1.9-fold increase in the E18.5 *Dermo1-Cre;Senp6*^*f/f*^ cartilage (Supplementary Figure 12), although its expression was not significantly changed in the RNA-seq results using cultured primary chondrocytes.

In addition, because p53 overactivity is one of the major mechanisms of organismal aging^26^, these results suggested that the premature aging phenotype seen in the *UBC-CreERT2;Senp6*^*f/f*^ *mice* was likely caused by the hyperactive p53. In support of this, we found by western blots that the tails from tamoxifen-treated *UBC-CreERT2;Senp6*^*f/f*^ mice had a distinctively higher amounts of total p53, p-p53, and SUMO2/3-modified proteins than those from control mice. In addition, the expression of p53 downstream target genes, such as *P21, Perp, Puma*, and *Pmaip1*, was significantly increased, similar to that observed in the *Dermo1-Cre;Senp6*^*f/f*^ cartilage (Supplementary Figure 13).

### SENP6 suppresses p53 activity by desumoylating TRIM28

Next, we studied how SENP6 regulates the p53 signaling pathway. p53 can be sumoylated at Lys386^27^, but the consequence remains unclear: sumoylation may enhance p53 exportation from the nucleus and degradation, or it may activate p53 by promoting its transactivity^28,29^. In our studies, we were unable to detect sumoylated p53 by western blots using either total cell lysates or a sumoylated protein-enriched fraction from WT and *Senp6*^*–/*–^ chondrocytes (Supplementary Figure 14). Hence, we sought to identify other SENP6 downstream targets that regulate p53 signaling. We enriched the total sumoylated proteins from *Dermo1-Cre;Senp6*^*f/f*^ and *Senp6*^*f/f*^ rib chondrocytes and profiled them using mass spectrometry. Our data showed that TRIM28 was one of the most abundant sumoylated proteins, with 3.2-fold enrichment in *Dermo1-Cre;Senp6*^*f/f*^ rib cartilage (Supplementary Figure 15).

**Fig. 5:**
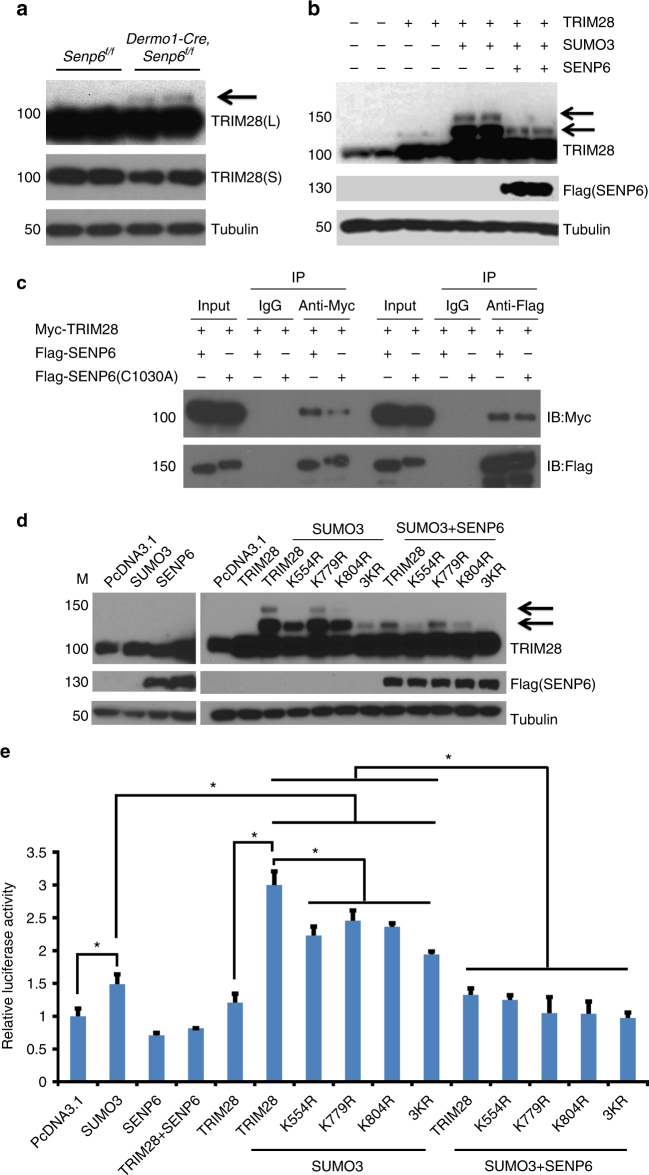
SENP6 desumoylated and stabilized TRIM28 and suppressed p53. **a** *Senp6* loss decreased the total TRIM28 protein in E18.5 *Dermo1-Cre;Senp6*^*f/f*^ rib cartilage but increased sumoylated TRIM28 (upper bands denoted by the arrow). “L” or “S” indicates long or short exposure (*n* = 3). **b** SUMO3 modifications on TRIM28 can be removed by overexpressed SENP6 (*n* = 2). **c** TRIM28 can be immunoprecipitated with either wild-type SENP6 or enzymatic-activity-null SENP6 (C1030A). **d**, **e**. Sumoylation-resistant TRIM28 mutations impaired TRIM28 sumoylation and p53 activation. Western blots showed that the K554R, K804R, K779R, and 3KR mutations of TRIM28 reduced TRIM28 SUMO3 modifications, and SENP6 effectively removed SUMO3 modification from all these sites (**d**). p53 reporter luciferase assays showed that the sumoylated TRIM28 enhanced p53 signaling, which was abolished by the addition of SENP6; these TRIM28 mutants induced significantly lower p53 activity than WT TRIM28 did (*n* = 3; error bars = standard deviation, *p < 0.05, Student’s *t*-test) (**e**)

TRIM28 is a multifaceted transcriptional co-repressor involved in chromatin remodeling, stem cell pluripotency, differentiation, DNA damage repair, and p53 regulation^30^. We found more sumoylated TRIM28 (arrow in Fig. 5a) but less total TRIM28 in the *Dermo1-Cre;Senp6*^*f/f*^ rib cartilage (Fig. 5a). In addition, overexpressed SENP6 effectively removed the SUMO3 modifications on TRIM28 (Fig. 5b). We further found that both WT SENP6 and the catalytic-domain-null SENP6 can be co-immunoprecipitated with TRIM28. These data suggested that a direct SENP6/TRIM28 interaction may mediate TRIM28 desumoylation (Fig. 5c). However, TRIM28 and p53 were not co-immunoprecipitated (Supplementary Figure 16), consistent with a previous report that TRIM28 does not regulate p53 activity by a direct interaction^31^.

Based on published results, TRIM28 has six known sumoylation sites, with three of them crucial to its functions (K554, K779, K804)^32,33^. To understand the preference of SENP6 in deconjugating SUMOs from these site(s), we overexpressed in HEK293 cells the TRIM28 variants with point mutations on each of these three sites (TRIM28^K554R^, TRIM28^K779R^, TRIM28^K804R^) or with the triple mutation (TRIM28^3KR^). Upon SUMO3 overexpression, TRIM28^3KR^ showed a strongest resistance to sumoylation (compared with WT TRIM28); TRIM28^K554R^ and TRIM28^K804R^ also had a distinctive decrease, while TRIM28^K779R^ showed a milder reduction in sumoylation. In addition, the overexpressed SENP6 effectively deconjugated SUMO3 modification from the WT and all these TRIM28 mutants (Fig. 5d). These data overall suggest that K554, K804, and K779 represented the major sumoylation sites of TRIM28, with the former two most predominant, and that SENP6 was capable of removing SUMO3 modifications from all these three sites.

Next, we evaluated the contribution of TRIM28 sumoylation to the p53 regulation by a reporter luciferase assay. We found that overexpression of SUMO3, TRIM28, or SENP6 alone caused very subtle changes of p53 activity. In contrast, SUMO3/TRIM28 co-overexpression induced a much stronger increase (~3 folds) of p53 activity, and this could be abolished by SENP6 overexpression. In addition, when SUMO3 was overexpressed, all the sumoylation-resistant TRIM28 mutants, especially the TRIM28^3KR^, showed less SUMO3 modifications and significantly weaker p53 reporter activity than the WT TRIM28 did (Fig. 5d, e). These data support the idea that SENP6-mediated TRIM28 desumoylation plays a crucial role in the regulation of p53 activity.

### Loss of p53 partially rescues skeletal defects of *Senp6*^*‒/‒*^ mice

**Fig. 6:**
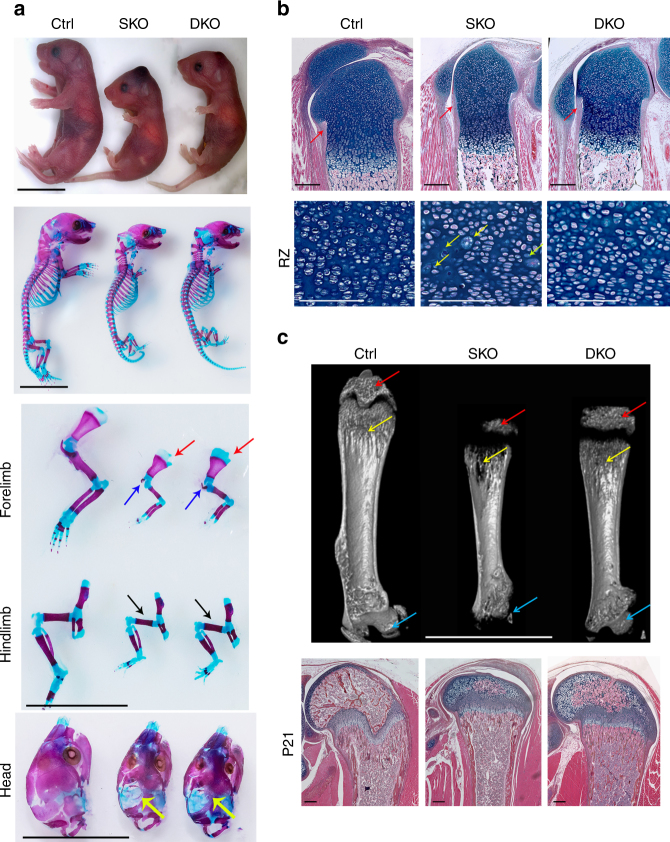
*Trp53* knockout partially rescued skeletal defects caused by *Senp6* loss. **a** *Prx1-Cre;Senp6*^*f/f*^*;Trp53*^*f/f*^ (DKO) P0 mice had a partially rescued skeletal phenotype relative to *Prx1-Cre;Senp6*^*f/f*^*;Trp53*^*f/+*^ (SKO) mice, as suggested by the smaller indentation in the scapula growth plate (red arrows), the longer clavicles (blue arrows), increased width and length of the femur (black arrows), tibia, humerus, ulna, and radius, and the moderately increased mineralization in the cranial bones (yellow arrows). Scale bars = 1 cm. **b** P0 DKO femoral sections show corrected growth plate diameter, perichondrium thickness (red arrows), and chondrocyte size (yellow arrows) relative to those of SKO mice. Scale bars = 100 μm for the upper panels and 50 μm for the lower panels. **c** p53 loss partially corrected the low bone mass and the delayed SOC formation of SKO mice at P21 as revealed by microCT imaging (SOC, red arrows; metaphasis bone, yellow arrows; proximal femur head, blue arrows; scale bars = 1 cm). H&E staining of the corresponding femurs is shown in the lower panels; scale bar = 100 μm

To directly test the hypothesis that the SENP6 desumoylase acts through the p53 axis in regulating bone development, we generated *Prx1-Cre;Senp6*^*f/f*^*;Trp53*^*f/f*^ mice (DKO). Relative to *Prx1-Cre;Senp6*^*f/f*^*;Trp53*^*f/+*^ (SKO) mice, DKO mice had an improved post-weaning survival rate (>60%), increased limb bone length and width, a thicker perichondrium, moderately corrected skeletal morphology and calvarial ossification, and partially rescued chondrocyte size (Fig. 6a, b). Most distinctively, the delayed SOC formation was well corrected (Fig. 6c). DKO mice at P21 showed clear cartilage canal formation in the SOC and a larger ossified area containing bone marrow in the epiphysis center, markedly exceeding those in the SKO mice. In addition, the DKO mice showed a higher trabecular bone mass in both the SOC and the metaphysis of distal femurs than SKO mice did (Fig. 6c). These data collectively suggest that excessive p53 signaling accounts for the skeletal defects in SKO mice.

**Fig. 7:**
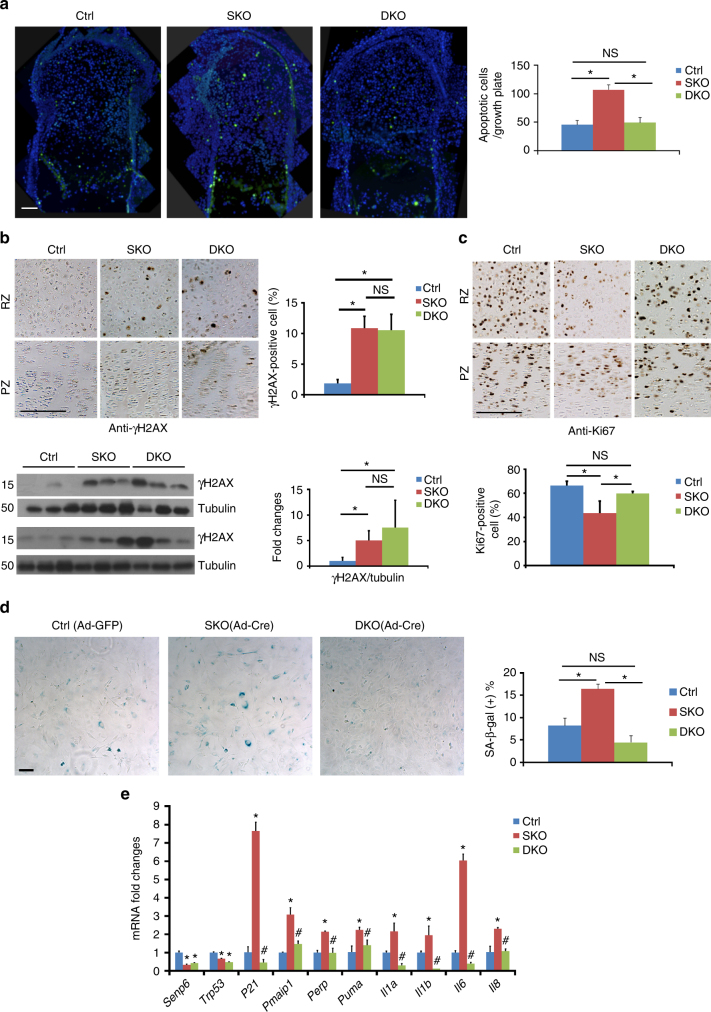
*p53* loss rescued the cellular phenotypes of SKO growth plates. **a** Growth plate of DKO mice shows decreased cell apoptosis, as indicated by TUNEL staining; the representative images are on the left and quantification on the right (*n* = 3; error bars = standard deviation, *p < 0.05; NS not significant, Student’s *t*-test; scale bar = 100 μm). **b** γH2AX expression was not rescued in the growth plate of DKO mice. The upper panel shows the representative IHC on the RZ and PZ of growth plate with quantification on the right; the lower panel shows western blots and quantification on the right (*n* = 6, error bars = standard deviation, *p < 0.05, NS not significant, Student’s *t*-test; scale bar = 50 μm). **c** Corrected cell proliferation by Ki67 staining in the growth plate of DKO mice relative to SKO mice (quantification shown in the low panel; *n* = 3, error bars = standard deviation, *p < 0.05, NS not significant, Student’s *t*-test; scale bar = 50 μm). **d** Corrected cell senescence in Ad-Cre-mediated-DKO vs SKO primary rib chondrocytes, as indicated by SA-β-gal staining; representative staining images are on the left and quantifications on the right (*n* = 3, error bars = standard deviation, *p < 0.05, NS not significant, Student’s *t*-test; scale bar = 50 μm). **e** Femur growth plate samples from DKO mice show the rescue of elevated p53 downstream targets as well as SASP markers (*n* = 3, *p < 0.05, relative to Ctrl; and ^#^*p* < 0.05, relative to SKO; error bars = standard deviation, Student’s *t*-test)

We then assessed whether *p53* deletion could rescue the defect in skeletal cell homeostasis caused by *Senp6* loss. Our data showed that DKO mice had significantly fewer apoptotic cells but more proliferating cells in the PZ and RZ of the growth plate. The γH2AX-positive cells in the growth plate of DKO mice were similar to those of SKO mice, and by western blot using growth plate protein samples we detected a moderately but insignificantly higher level of γH2AX in DKO than in SKO (Fig. 7a, c). DKO primary rib chondrocytes showed less SA-β-gal staining than did SKO and control chondrocytes (Fig. 7d). Also, the DKO calvarias and femur growth plates had decreased expression of p53 downstream genes and SASP genes (Fig. 7e, Supplementary Figure 17), suggesting that the elevated p53 signaling in *Senp6*^*‒/‒*^ chondrocytes not only promoted apoptosis and cell senescence but also enhanced SASP. This is in line with the previous finding that p53 promotes SASP in the liver cells^34^. Consistently, *Senp6*^*‒/‒*^ BMSCs were more susceptible to the cell senescence induced by nutlin-3 (a p53 activator) than were control (*Senp6*^*f/f*^) cells. In contrast, pifithrin-α (PFTα, a p53 inhibitor) was more efficient in rescuing the senescence *of Senp6*^*‒/‒*^ BMSCs, relative to *Senp6*^*f/f*^ BMSCs. This suggests that excessive p53 signaling contributes to the advanced cell senescence of *Senp6*^*‒/‒*^ cells (Supplementary Figure 18).

## Discussion

Sumoylation regulates the functions of a wide variety of proteins associated with human diseases^35^. However, the role of the sumoylation/desumoylation pathways has not been well studied in skeletal development and aging. In the present work, we found that global deletion of *Senp6* in adult mice caused a distinctive premature-aging phenotype. OCP-specific *Senp6* deletion resulted in a broad range of skeletal developmental defects associated with enhanced apoptosis and cell senescence. In addition, *Senp6* loss markedly elevated inflammatory and tissue-degrading factors of SASP components in the chondrocytes and OCPs. These observations suggest that SENP6 plays a role in preventing skeletal aging by inhibiting both stem cell/progenitor exhaustion and abnormal cell–cell communication, the two ultimate hallmarks of aging^3^. It has been reported that a decreased SENP6 level and an SNP adjacent to the *SENP6* locus are associated with osteoarthritis^13,14^, which is triggered and aggravated by articular chondrocyte senescence and the SASP. Whether and how SENP6 plays a role in joint aging and in the pathogenesis of osteoarthritis is worthy of further study.

Our work revealed that *Senp6* loss significantly up-regulated p53 signaling. The p53 pathway promotes cell senescence and apoptosis, as well as organismal aging^36^. Mice with a constitutively active p53 mutation (*Trp53*^*TSD*^) exhibit severe premature aging with kyphosis^37^, similar to the *UBC-CreERT2;Senp6*^*f/f*^ mice. Moreover, a deficiency of MDM2 (an E3 ubiquitin ligase destabilizing p53) in mesenchymal cells or OCPs produces a smaller skeleton and cranial defects^38^, reminiscent of the *Dermo1-Cre;Senp6*^*f/f*^ or *Prx1-Cre;Senp6*^*f/f*^ mice. We showed that p53 loss can partially but distinctively rescue these *Senp6*-loss-induced skeletal phenotypes. These results support the idea that the p53 is an important downstream pathway of SENP6 in regulating skeletal development and maintenance. In addition, as a major downstream target of p53, p21 plays a pivotal role in promoting cell senescence. Excessive p21 expression is commonly observed in senescent cells and aging animal models, and the removal of p21 can alleviate cell senescence^36^. We observed a dramatic up-regulation of p21 in *Senp6*-deficient cells, and further removal of p53 effectively normalized p21 expression and the cell senescence phenotype, implying an important contribution of the p53–p21 axis in *Senp6*-loss-induced cell senescence phenotype (Fig. 7e, Supplementary Figure 17). Moreover, the well-rescued SOC formation in *Prx1-Cre;Senp6*^*f/f*^ mice by p53 loss suggested an unprecedented role of p53 signaling in regulating SOC formation, i.e., SOC initiation requires a low local p53 activity.

We showed that SENP6 loss led to increased TRIM28 sumoylation but a decreased total TRIM28 in chondrocytes and OCPs, in line with the findings that TRIM28 is a negative regulator of p53 and cellular senescence. For example, TRIM28 has been shown to promote p53 degradation through MDM2- or MAGEs-dependent pathways^31,39,40^. Also, TRIM28 was reported to be a major target of senescence-associated protein degradation (SAPD), and its loss can trigger cellular senescence in primary fibroblasts^41^. Moreover, sumoylation promotes TRIM28 degradation by RNF4, a sumoylation-dependent ubiquitin E3 ligase^42^. These results suggest that SENP6 may partly prevent p53-hyperactivity-caused cellular senescence by desumoylating and stabilizing TRIM28. Moreover, our luciferase reporter assay showed that the SUMO3 modifications of overexpressed TRIM28 could augment p53 activity, which could be further negated by SENP6 overexpression (Fig. 5e). This suggests that TRIM28 sumoylation could also enhance p53 pathway in a degradation-independent manner. However, these findings apparently differ from previous reports: TRIM28 and sumoylated TRIM28 have been reported to be transcriptional co-repressors of ZBRK1, a zinc-finger DNA-binding protein that mediates transcriptional repression of a few p53 downstream genes^43^; when ZBRK1 is overexpressed, the N-terminal SUMO1-TRIM28 in-frame fusion protein can suppress doxorubicin-induced *p21* promoter activation in the MCF-7 breast cancer cell line^44^. The discrepancies between these two studies and our result may be due to the reason that our work explored the general role of SUMO3-modified TRIM28 sumoylation/desumoylation on p53 activation, while the two studies above focused on ZBRK1-dependent TRIM28 function.

Besides TRIM28, SENP6 has been found to interact with and desumoylate other p53 regulators, such as TIP60 and PML^45,46^. However, PML and TIP60 did not appear in the results of our SUMO IP/MS experiment; their roles in mediating the *Senp6*-loss-induced p53 activation in the mesenchymal cell lineage remain unclear.

We found that *Senp6*^*‒/‒*^ chondrocytes had an impaired capacity in clearing irradiation-induced DNA damage. This is consistent with the reports that SENP6 interacts with and desumoylates the RPA70 and Fanconi Anemia ID complex and thus facilitates replication fork processing during DNA repair^15,16^. In support of this, we also found that the level of RPA70 sumoylation was augmented in *Senp6*-deficient cartilage (Supplementary Figure 9). As a pivotal regulator of DNA damage repair, p53 is quickly activated in response to DNA damage^47^. The dramatically enhanced p53 activity and cell senescence in the *Senp6*-deficient skeletal cells may be partly due to a prolonged stress response to the impaired DNA repair. However, in HEK293 cells, TRIM28/SUMO3 co-overexpression did not seem to alter the level of γH2AX (Supplementary Figure 19), while it was still sufficient to enhance p53 reporter activity (Fig. 5e). This implies that, at least in such an experimental setting, the SENP6–TRIM28 axis could regulate p53 pathway in a DNA damage-independent manner. Interestingly, we found that *p53* loss partially rescued the skeletal phenotype and cell senescence caused by *Senp6* deficiency, but it did not rescue (or even worsen) the DNA damage. This phenomenon is reminiscent of several previous reports: *Trp53* loss can rescue the premature aging and cell senescence phenotypes of *Brca1* or *Ku80* knockout mice at the expense of increasing DNA damage^48,49^.

We showed that p53 loss resulted in a distinct but incomplete rescue of *Senp6*-KO phenotypes, implying that other p53-independent pathways may also contribute to the regulation of SENP6-mediated OCP homeostasis. Moreover, TRIM28 and its sumoylated forms have been found to orchestrate chromatin remodeling by modulating DNA and histone modifications^50,51^. In support of this, a recent finding showed that both TRIM28 and SENP6 are important factors in suppressing endogenous retrovirus activation, which is commonly implemented through chromatin silencing^52^. How the SENP6/TRIM28 interaction epigenetically regulates stem cells/progenitor dynamics is a promising direction for further exploration.

Interestingly, SENP7, another member of desumoylase family, has also been found to modulate TRIM28 function in chromatin relaxation, which is an important biological event for DNA damage repair and epigenetic regulation^53^. Our RNA-seq data showed that SENP7 (RPKM = 4.85) was expressed at a relatively lower level than SENP6 (RPKM = 14.4), and its expression was unaltered in the BMSCs of aging mice and unaffected by *Senp6* loss in the chondrocytes (Fig. 1b, Supplementary Figure 11). Also, desumoylase SENP5 has been reported to regulate DNA repair and mitochondrial function, both of which are highly associated with aging^54,55^. We found that *Senp5* expression was decreased in the BMSCs of aging mice (Fig. 1b), but its expression was not affected by *Senp6* loss in the chondrocytes (Supplementary Figure 11). It remains unclear whether SENP7 and SENP5 have a redundant role of SENP6 in the skeletal context.

Overall, our study suggests that during skeletal development, SENP6 desumoylase maintains proper OCP renewal, differentiation, and survival, partly through suppressing p53 pathways. These findings will shed new light into research in fields such as cell senescence, stem cell rejuvenation, aging, and cancer, where sumoylation and p53 pathways play crucial roles.

## Methods

### Mice

The *Dermo1-Cre*, *Prx1-Cre*, *UBC-CreERT2, and Trp53*^*f/f*^ mice were obtained from Jackson Lab and maintained by breeding with WT C57BL/6 strains. The *Senp6* floxed mice were created by the laboratory of Dr. Edward Yeh and maintained on C57BL/6 background. Mice at embryonic or perinatal stages were not separated by sexes. The mice at p12, p21, and adult age used in the present study were males. The littermates were randomly grouped based on genotypes for all the experiments. The animal procedures were approved by the Van Andel Research Institute Animal Care and Use Committee.

### Skeletal staining

The scarified mice with skin and internal organs removed were fixed in 95% ethanol overnight and stained with alcian blue (0.3% in 70% ethanol) for 24–48 h. Next, the skeletons were incubated with 2% KOH for 24–48 h, then stained with alizarin red S (0.1% in 95% ethanol) overnight. The stained skeletons were cleared in 1% KOH/20% glycerol solution for 2–3 days, and stored in ethanol/glycerol solution (1:1).

### Histology and immunohistochemistry

For histological analyses, embryonic undecalcified or adult decalcified (using 14% EDTA solution) mouse skeletal tissues were fixed in 4% paraformaldehyde and were paraffin-embedded. Specimens were sectioned at a thickness of 5 µm. Sections were stained with Alcian blue hematoxylin/orange G following standard procedures.

For immunohistochemistry, sections were dewaxed and rehydrated, antigen-unmasked in citrate buffer, and then were quenched for endogenous peroxidase activity using 3% H_2_O_2_ for 10 min. After rinsing in PBS, the sections were incubated in the normal serum blocker (NSB) buffer (3% normal serum, 0.1% BSA, and 0.1% Triton X-100 in PBS) for 30 min and then were reacted with specific primary antibodies overnight at 4 °C. After washing, sections were incubated with biotinylated secondary antibody for 1 h at room temperature, treated with avidin–biotin–peroxidase complex reagent (ABC, Vector Laboratories) for 30 min at room temperature, followed by incubation with peroxidase substrate solution (DAB) (Vector Laboratories). Finally, sections were counterstained with hematoxylin for 20 s and mounted with coverslips^56,57^. Detailed information about the antibodies used in this study is provided in Supplementary Table 1.

### microCT imaging

Following the JBMR-recommended microCT guidelines and previous publications^58,59^, we scanned the P21 male mouse femurs at 13.3-µm voxel size (59 kV, 167 μA) using a SkyScan 1172 microCT imaging system. After reconstruction using the RNecon software with a threshold set at 90 and aligned in the Dataviewer software, 3D images were created using the CTan software. The sagittal section views of femurs were captured using the CTvol software.

### BrdU incorporation and TUNEL assays

For the cell proliferation assay, BrdU labeling reagent (10 µl/g body weight; Life Technologies) was injected into pregnant female mice or postnatal mice. Two hours later, the mice were sacrificed, and the growth plate samples were collected for sectioning. The tissue sections were dewaxed and permeabilized with 0.5% Triton X-100:PBS, blocked in NSB buffer, and stained with anti-BrdU antibody (Life Technologies) and anti-mouse AlexaFluor-488 secondary antibody (Molecular Probes). Mounting medium containing DAPI was used to counterstain nuclei. Images were acquired with a 20× objective and the positive staining was quantified manually using Image-Pro-Plus 6.0 software^60^.

The TUNEL assay was performed on paraffin-embedded sections of distal femur growth plates using an in situ Cell Death Detection Kit (Roche Diagnostics) according to the manufacturer’s instructions. For quantification, the number of TUNEL-positive cells was counted in a section of the entire distal femur growth plate for each sample.

### SA-β-gal staining assay

The activity of SA-β-gal in cell cultures was detected according to the reported protocols^61^. Briefly, cultured cells were fixed by 0.5% glutaraldehyde, washed with PBS, then incubated with 1 mg/ml X-gal in SA-β-gal staining solution (5 mM potassium ferrocyanide, 5 mM potassium ferricyanide, and 2 mM MgCl_2_, pH 6.0) at 37 °C overnight. For frozen sections, SA-β-gal activity was determined by using an SA-β-gal kit (Cell Signaling Technology) according to the manufacturer’s instructions.

### Cell culture

Primary mouse rib chondrocyte cultures: Rib cages were dissected from E18.5 mouse embryos. The soft and bony tissues surrounding the cartilaginous parts of the rib were removed as much as possible. Rib cages were incubated in pronase (2 mg/ml in PBS) at 37 °C for 30 min, followed by incubation in collagenase D (3 mg/ml in DMEM without serum) at 37 °C for 30–60 min to further remove soft tissue. Then the cleaned rib cartilage pieces were transferred into 1.5-ml centrifuge tubes and incubated in collagenase D digestion solution (DMEM with 1.5 mg/ml collagenase D and 5% fetal bovine serum (FBS)) overnight at 37 °C. The dissociated chondrocytes were collected and plated in α-MEM medium with 10% FBS.

BMSC cultures: Bone marrow was flushed out from mouse tibia and femurs and was cultured in α-MEM with 10% FBS. After 48 h, non-adherent cells were removed by three washes in PBS and the adherent cells (i.e., BMSCs) were continuously cultured with medium changes every 2 days. After 7–10 days in culture, BMSCs were subcultured. All experiments were performed using cells at the second or third passage if not specified. To induce differentiation and mineralization, BMSCs were cultured in a mineralization medium (growth medium plus 50 µg/ml ascorbic acid, 1 mM β-glycerophosphate, and 0.01 µM dexamethasone).

CFU-F and CFU-O cultures: The total bone marrow was harvested from mouse femurs and tibias, seeded at 1 × 10^6^ cells/well in 12-well plates, cultured for 48 h, and then washed to remove unattached cells. For the CFU-F (fibroblast) assay, the cells were cultured for another 8 days in normal culture medium (α-MEM, 10% FBS) and then were stained with crystal violet. For the CFU-O (osteoblast) assay, the cells were cultured for another 8 days in mineralization medium and then stained for alkaline phosphatase (ALP)^59^.

The HEK293 cell line was obtained from ATCC and was cultured in DMEM medium with 10%FBS.

### Western blots

Total protein was extracted from mouse rib cartilage or from cell cultures using RIPA buffer. Samples containing equal amounts of protein were separated by SDS-PAGE gel and subjected to standard western blot procedures. Antibodies against SUMO1, SUMO2/3 were purchased from Sigma; anti-RPA70 was purchased from Santa Cruz; anti-p53, anti-p-p53, anti-γH2AX, anti-TRIM28, and anti-α-tubulin antibodies were purchased from Cell Signaling Technology. The original uncropped scans of western blots are shown in Supplementary Figure 20, 21. Detailed information about the antibodies used in this study is provided in Supplementary Table 1.

### Co-immunoprecipitation

HEK293 cells were transfected with Myc-tagged TRIM28, 3XFlag-SENP6, or 3XFlag-SENP6(C1030A)^15^ plasmids using X-fect transfection reagent (Clontech Laboratories). After culturing for 48 h, cells were lysed in RIPA buffer containing protease inhibitor and NEM (*N*-ethylmaleimide). Myc (Invitrogen, 1:1000) or Flag (Sigma-Aldrich, 1:1000) antibodies were used to immunoprecipitate TRIM28 and SENP6 or the SENP6 mutant (C1030A), respectively. The precipitates were boiled in Laemmli buffer (Bio-Rad) with 5% β-mercaptoethanol and loaded onto a 6% PAGE-SDS gel for western blot assays.

### Quantitative RT-PCR (qRT-PCR)

Total RNA was extracted form mouse rib cartilage, calvaria, or cell cultures using TRIzol (Invitrogen), followed by use of a GenElute Mammalian Total RNA kit (Sigma) with on-column DNase digestion. First-strand cDNA was generated from 1 μg of RNA using SuperScript III First-strand Synthesis System (Invitrogen). Real-time PCR was performed using the StepOnePlus system and Fast SYBR Green Master Mix (Invitrogen). The relative gene expression levels were calculated using 2^–ΔΔCT^ method^62^. The actin gene was used as an internal expression control. The specific primer sequences used in the study are provided in Supplementary Table 2.

### γH2AX foci assays

X-ray irradiations were performed at 225 kVp; dosage was 2 Gy at a rate of 0.4 Gy/min hardened with a 4-mm removable copper filter^63^. To minimize variations, radiation exposures were conducted at room temperature with cells covered in 2 ml of warmed culture medium. Pre- and post-irradiation, cells were immediately transferred to a 37 °C, 5% CO_2_ incubator in the same room. Control experiments were performed following exactly the same irradiation protocol but without energizing the X-ray unit. After irradiation, cells were fixed at different time points for plotting the kinetics of γ-H2AX induction and clearance. Cells were fixed in ice cold 4% paraformaldehyde for 15 min at room temperature, permeabilized with 0.5% Triton X-100:PBS, blocked in NSB buffer (Upstate), and stained with anti-γ-H2AX antibody (Upstate) and anti-mouse AlexaFluor-488 secondary antibody (Molecular Probes). Mounting medium containing DAPI was used to counterstain nuclei. Images were acquired with a 100× objective. The γ-H2AX foci were scored manually using Image-Pro-Plus 6.0 software, and the average number of foci per cell was calculated from a minimum of 200 cells per time point. Experimental data represent the average of two independent experiments.

### RNA sequencing and data analysis

Total RNA was extracted from the primary chondrocytes of *Dermo1-Cre;Senp6* ^*f/f*^ and *Senp6* ^*f/f*^ mice rib cartilage using GenElute Mammalian Total RNA Miniprep Kit (Sigma) according to the manufacturer’s instructions. RNA was then sequenced by the BGI RNA-seq service (*n* = 2). GO analysis was performed using DAVID (http://david.abcc.ncifcrf.gov/). Gene expression clustering was analyzed using Cluster 3.0 and visualized using Java TreeView.

For Gene Set Enrichment Analysis, we used GSEA v2.2.0 on various functional and/or characteristic gene signatures. Gene sets were obtained from the MSigDB database v3.0. Statistical significance was assessed by comparing the enrichment score to enrichment results generated from 1000 random permutations of the gene set to obtain *p* values (nominal *p* value).

### LC/MS/MS

Sumoylated proteins were enriched from the primary chondrocytes using a SUMO-QAPTURE-T Kit (Enzo-Lifesciences) following the manufacturer’s protocol and were analyzed at the Mass Spectrometry Core Facility of Michigan State University. Briefly, samples were purified and concentrated in a one-dimensional (1D) SDS-PAGE gel, and the gel bands were dehydrated and then digested using sequencing grade-modified trypsin. Peptides were extracted from the gel by water bath sonication and then re-suspended. Eluted peptides were sprayed into a ThermoFisher Q-Exactive mass spectrometer using a FlexSpray spray ion source. Survey scans were taken in the Orbi trap (70,000 resolution, determined at *m*/*z* 200) and the top 10 ions in each survey scan were then subjected to automatic higher energy collision–induced dissociation (HCD) with fragment spectra acquired at 17,500 resolution. The resulting MS/MS spectra were converted to peak lists using Mascot Distiller, v2.5.1, and searched against all mouse protein entries available from the UniProt database appended with common laboratory contaminants and from the GPM, using the Mascot searching algorithm, v2.5. The Mascot output was then analyzed using Scaffold v4.4.6 to probabilistically validate protein identifications. Assignments validated using the Scaffold 1% FDR confidence filter was considered true. After filtering non-specific hits, proteins having at least two peptides detected by MS and more than a 2.2-fold increase in the *Dermo1-Cre;Senp6*^*f/f*^ chondrocytes relative to controls were considered candidate substrates of SENP6.

### p53 reporter assay

HEK293 cells (ATCC) were transfected with PG13-Luc (with p53 binding cis-element, 0.5 μg) and Myc-TRIM28, TRIM28^K554R^, TRIM28^K779R^, TRIM28^K804R^, TRIM28^3KR^ (0.5 μg) with HA-SUMO3 (0.5 μg) or Flag-SENP6 (1.5 μg) present or absent based on the experimental strategy. Each transfection included Flag-p53 (0.05 μg) and pRL-Renilla (0.05 μg, as control). After incubation for 48 h at 37 °C, the cell lysate was collected for luciferase assay using a Dual-Luciferase kit (Promega). The firefly-luminescence results were normalized to the Renilla luminescence for internal control of transfection efficiency.

### Statistical analysis

All statistical results are presented as the mean ± standard deviation (S.D.). The differences between two groups were calculated using the two-tailed, unpaired Student’s *t*-test; *p* values <0.05 were considered statistically significant. If not specified in the figure legends, the number of animals per group was *n *≥ 3 for the in vivo experiments, including macroscopic phenotyping, skeletal staining, histology, immunohistochemistry, genetic rescues, qRT-PCR, and western blots. The representative images were those in good agreement with the consistent observations. For the in vitro or ex vivo studies, the number of biological replicates (*n*) is indicated in each figure legend.

### Data availability

RNA-seq data that support the findings of this study have been deposited in Gene Expression Omnibus with the accession codes (GSE102856). All relevant data are available from the authors upon reasonable request.

## Electronic supplementary material

Supplementary Information
