# Publisher Correction: Desumoylase SENP6 maintains osteochondroprogenitor homeostasis by suppressing the p53 pathway

**DOI:** 10.1038/s41467-018-03177-0

**Published:** 2018-02-08

**Authors:** Jianshuang Li, Di Lu, Hong Dou, Huadie Liu, Kevin Weaver, Wenjun Wang, Jiada Li, Edward T. H. Yeh, Bart O. Williams, Ling Zheng, Tao Yang

**Affiliations:** 1 Center for Cancer and Cell Biology, Van Andel Research Institute, Grand Rapids, MI 49503 USA; 2 Hubei Key Laboratory of Cell Homeostasis, College of Life Sciences, Wuhan University, Wuhan, 430072 Hubei China; 3 Center for Precision Medicine, Department of Medicine, University of Missouri, Columbia, MO 65212 USA; 4 State Key Laboratory of Medical Genetics and School of Life Sciences, Central South University, Changsha, 410078 Hunan China

Correction to: *Nature Communications* 10.1038/s41467-017-02413-3, published online 2 November 2017

In the originally published version of this Article, the positions of the final two authors in the author list were inadvertently inverted during the production process. This error has now been corrected in both the PDF and HTML versions of the Article.

