# Supplementary material for: Desumoylase SENP6 maintains osteochondroprogenitor homeostasis by suppressing the p53 pathway
Source: Nat Commun. 2018 Jan 10;9:143. doi: 10.1038/s41467-017-02413-3 (PMC5762923; doi:10.1038/s41467-017-02413-3)
Supplement: Supplementary file 1 — Supplementary Information [file 41467_2017_2413_MOESM1_ESM.pdf]

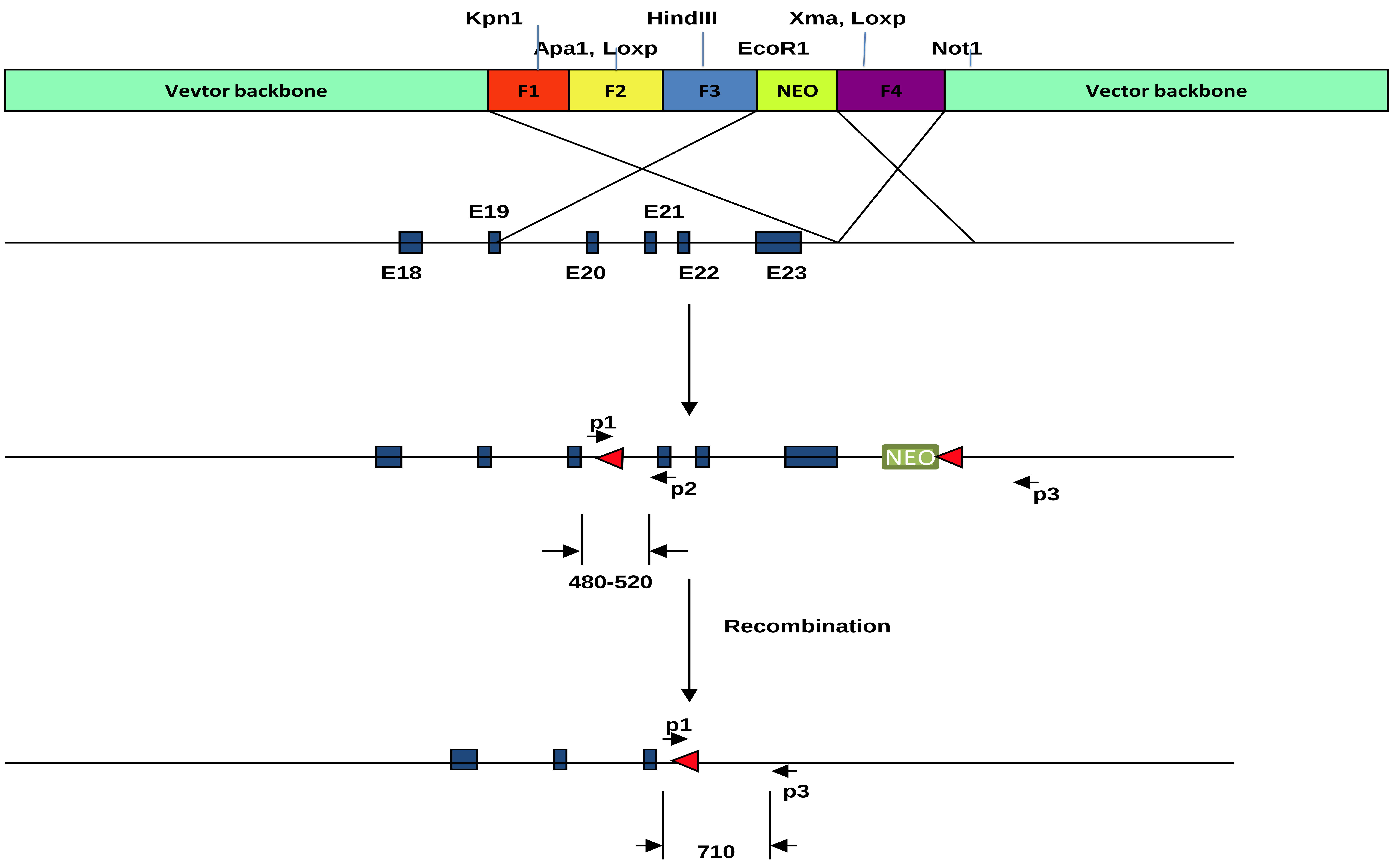

Supplementary Figure 1. Schematics of the *Senp6* floxed allele. Two *loxP* sites were inserted into intron 19 and intron 23 of *Senp6* to generate a *Senp6* floxed allele. The restriction enzyme sites for cloning and primers for genotyping the WT (P1+P2, 480 bp), floxed (P1+P2, 520 bp), and deleted (P1+P3, 710 bp) alleles are shown.

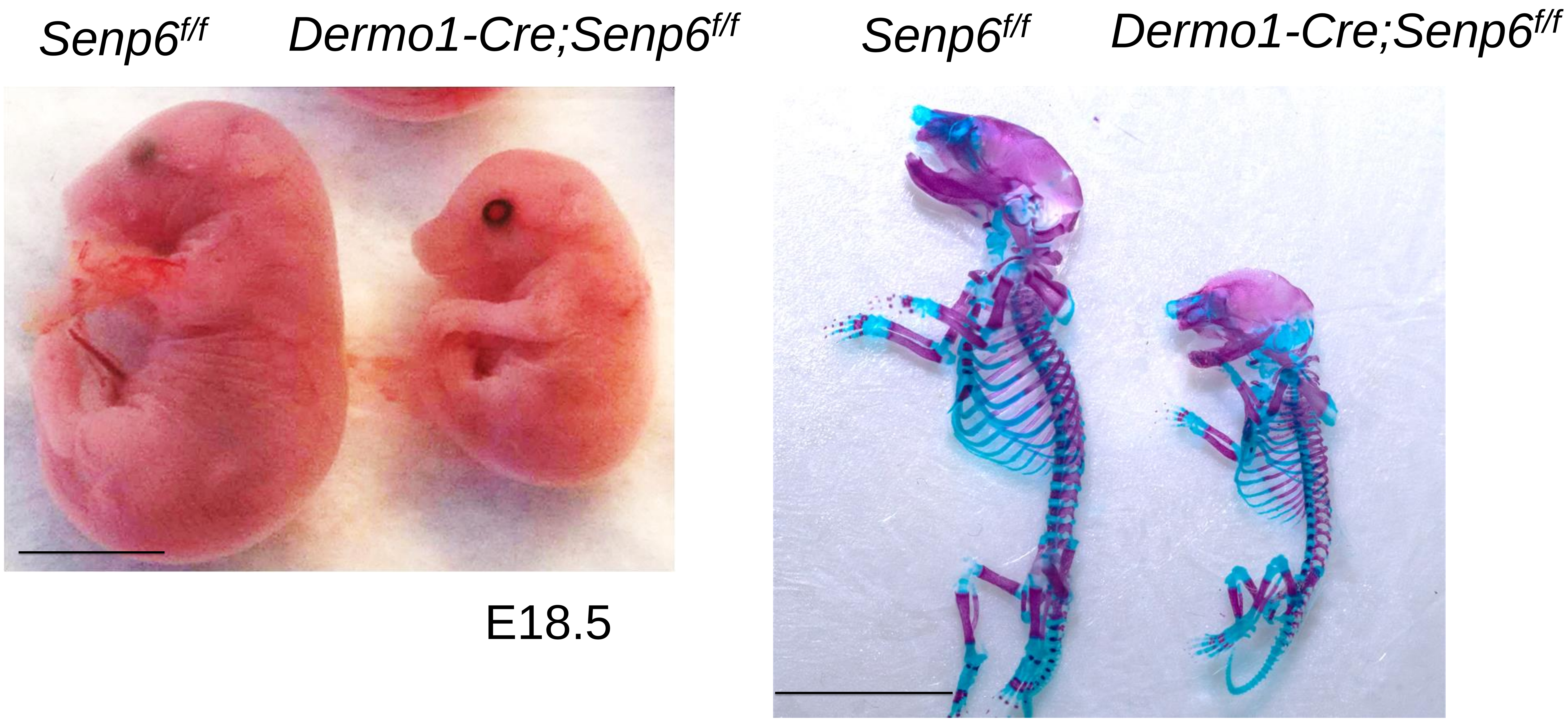

Supplementary Figure 2. E18.5 *Dermo1-Cre;Senp6*$^{f/f}$ mice had a smaller body (left) and skeleton (right) than control littermates (*Senp6*$^{f/f}$). Scale bars = 1 cm.

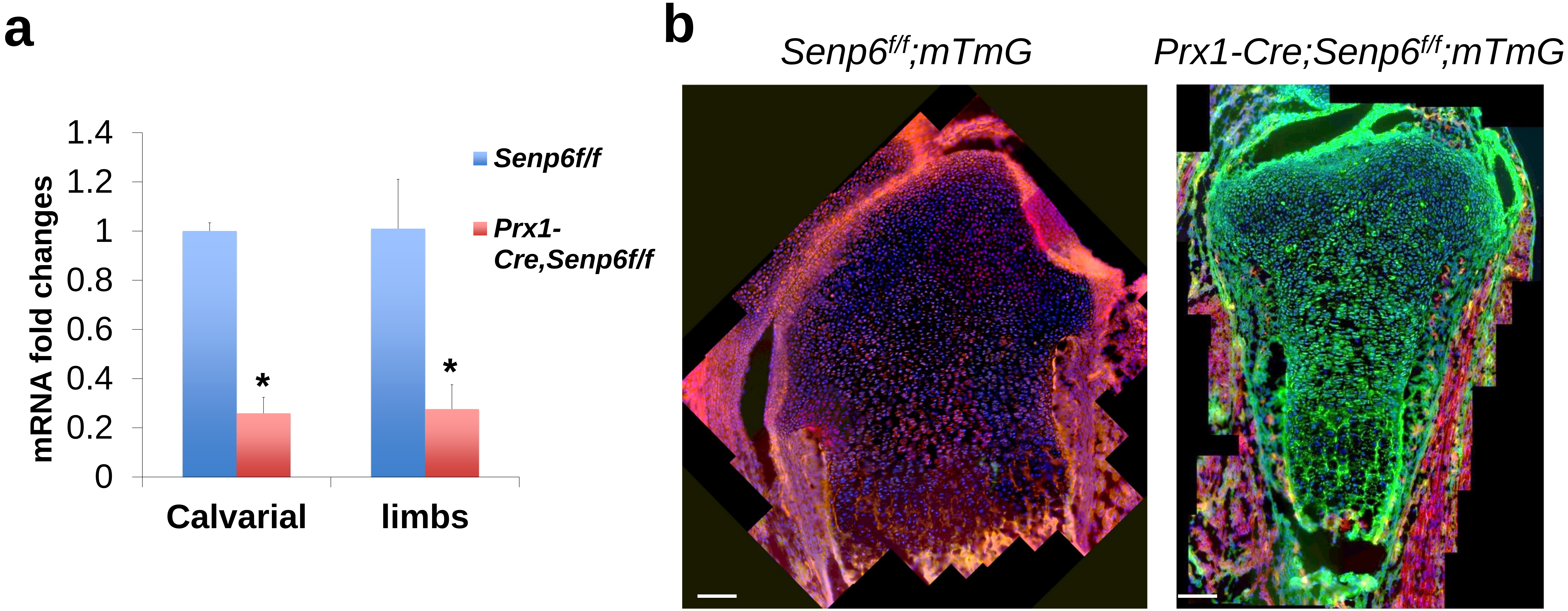

Supplementary Figure 3. a. *Prx1-Cre* alleles can effectively delete the *Senp6* gene in mouse skeletal tissues (n = 3, error bars = standard deviation, $^{*}p<0.05$, compared to control mice, Student's t-test). **b**. *Prx1-Cre* leads to high penetrance of gene deletion in the growth plate and perichondrium, as indicated by the GFP expression caused by Cre-mediated recombination in *mTmG* allele (scale bars = 100 μm).

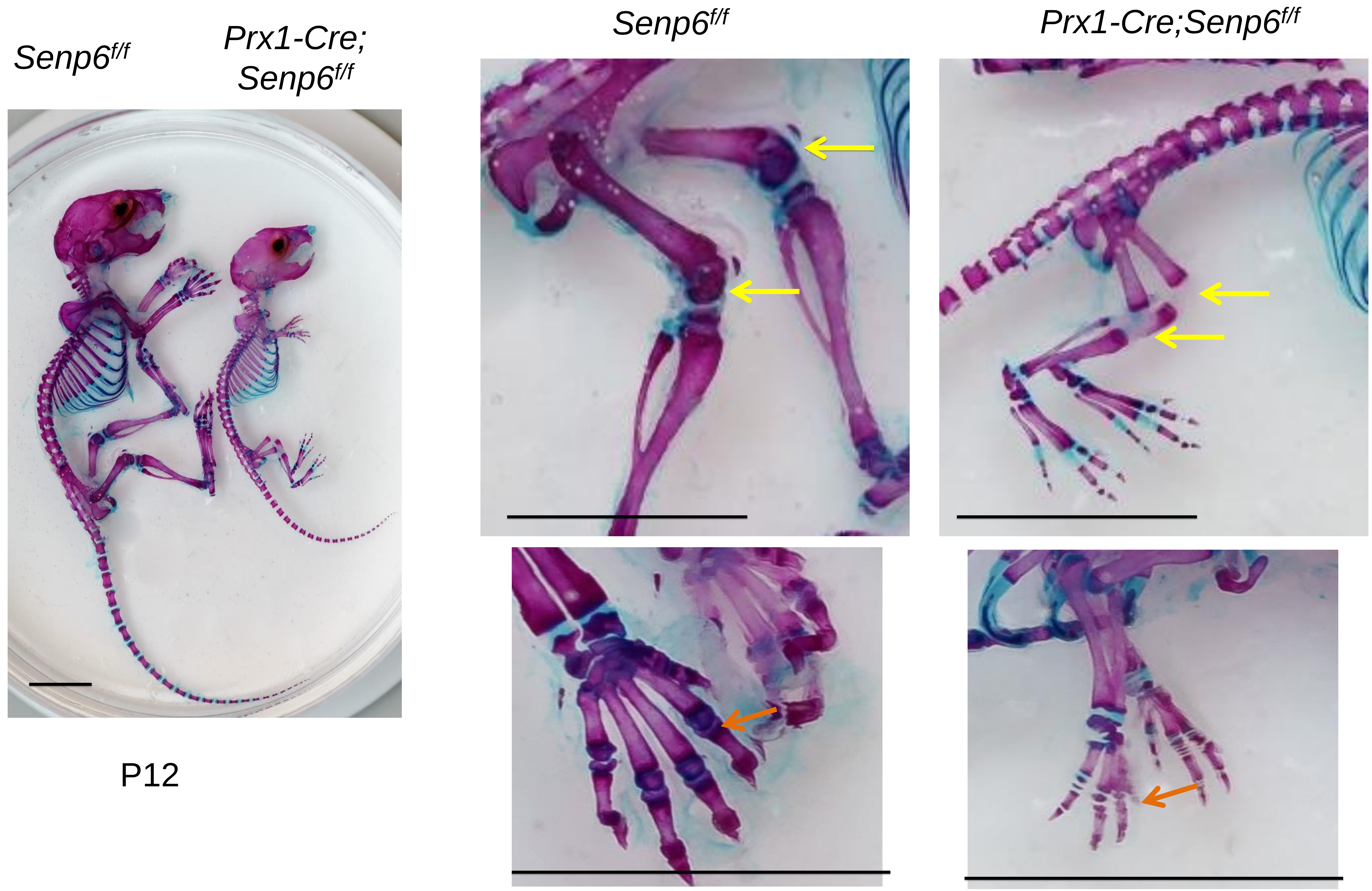

Supplementary Figure 4. P12 *Prx1-Cre;Senp6*$^{f/f}$ mice show undermineralized epiphysis (lack of mineralized SOC, yellow arrows), and missing metacarpals, (i.e., unseparated 2nd and 3rd metacarpals attached to the 2nd and 3rd phalanges, orange arrows). (scale bars = 1 cm).

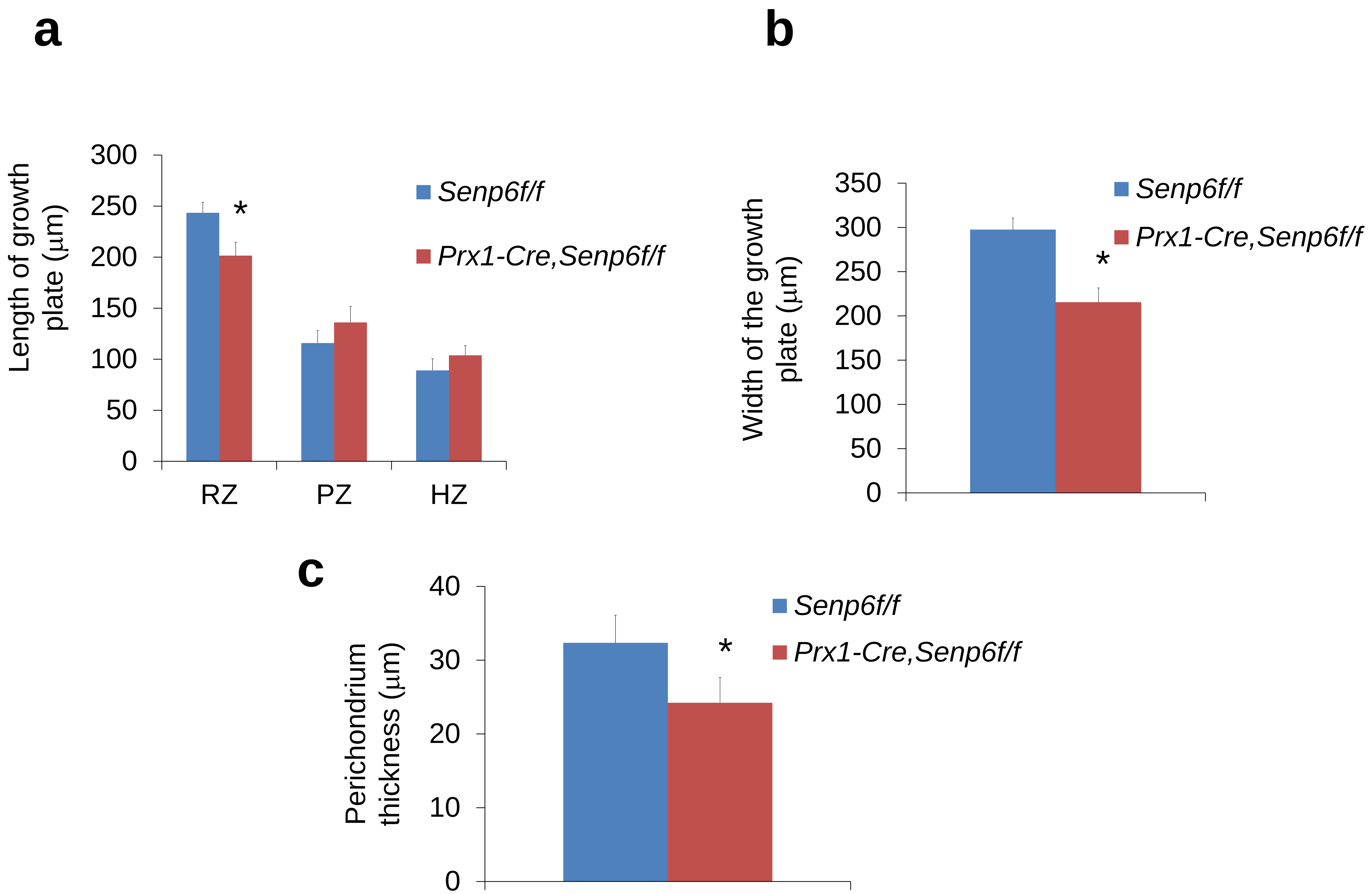

Supplementary Figure 5. P0 *Prx1-Cre;Senp6*$^{f/f}$ mice showed decreased length (**a**), width (**b**), and perichondrium thickness (**c**) of the growth plate. (n = 3**,** error bars = standard deviation, *p<0.05, compared to control mice, Student's t-test) .

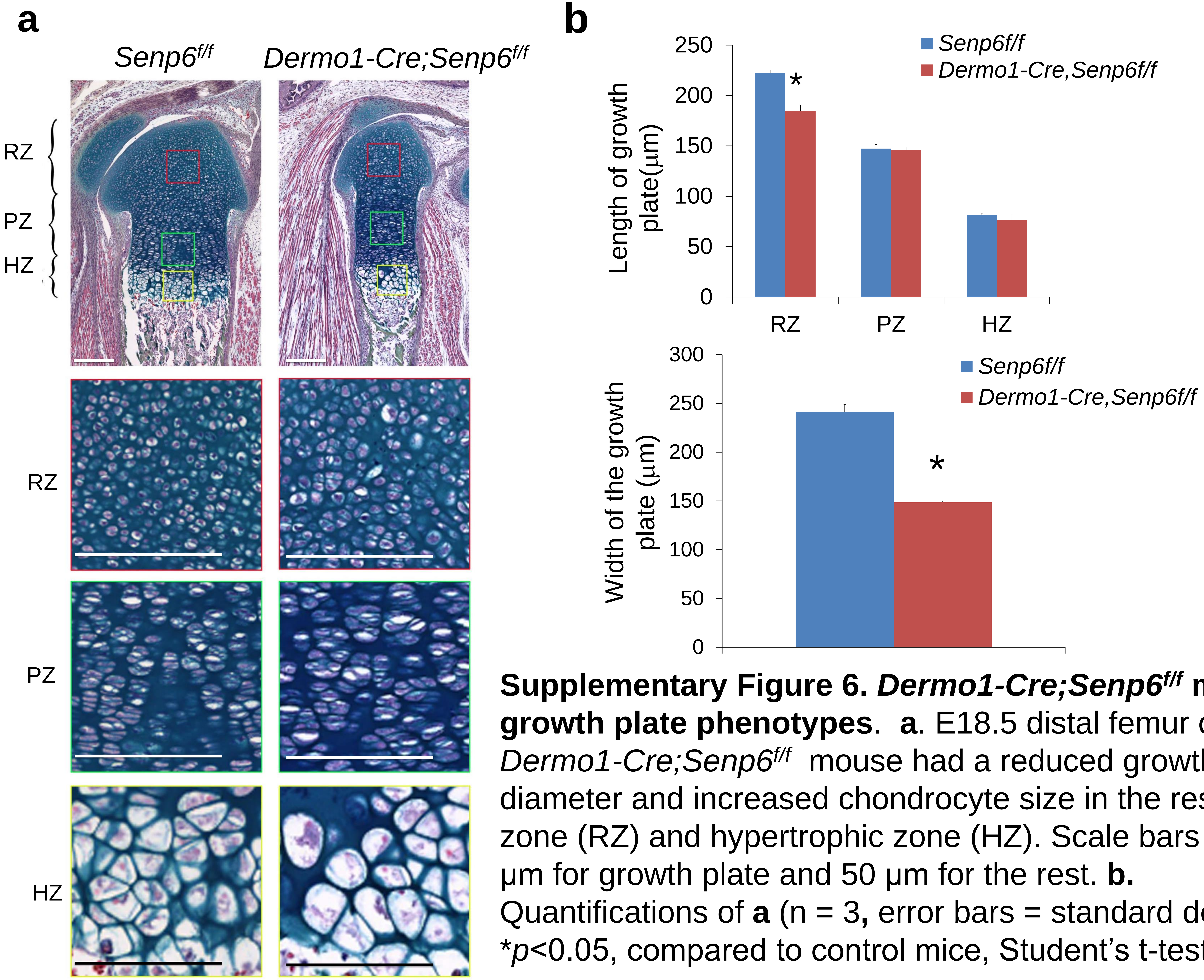

Supplementary Figure 6. *Dermo1-Cre;Senp6$^{f/f}$* mice growth plate phenotypes. **a**. E18.5 distal femur of *Dermo1-Cre;Senp6$^{f/f}$* mouse had a reduced growth plate diameter and increased chondrocyte size in the resting zone (RZ) and hypertrophic zone (HZ). Scale bars = 100 μm for growth plate and 50 μm for the rest. **b.** Quantifications of **a** (n = 3**,** error bars = standard deviation, **p*<0.05, compared to control mice, Student's t-test).

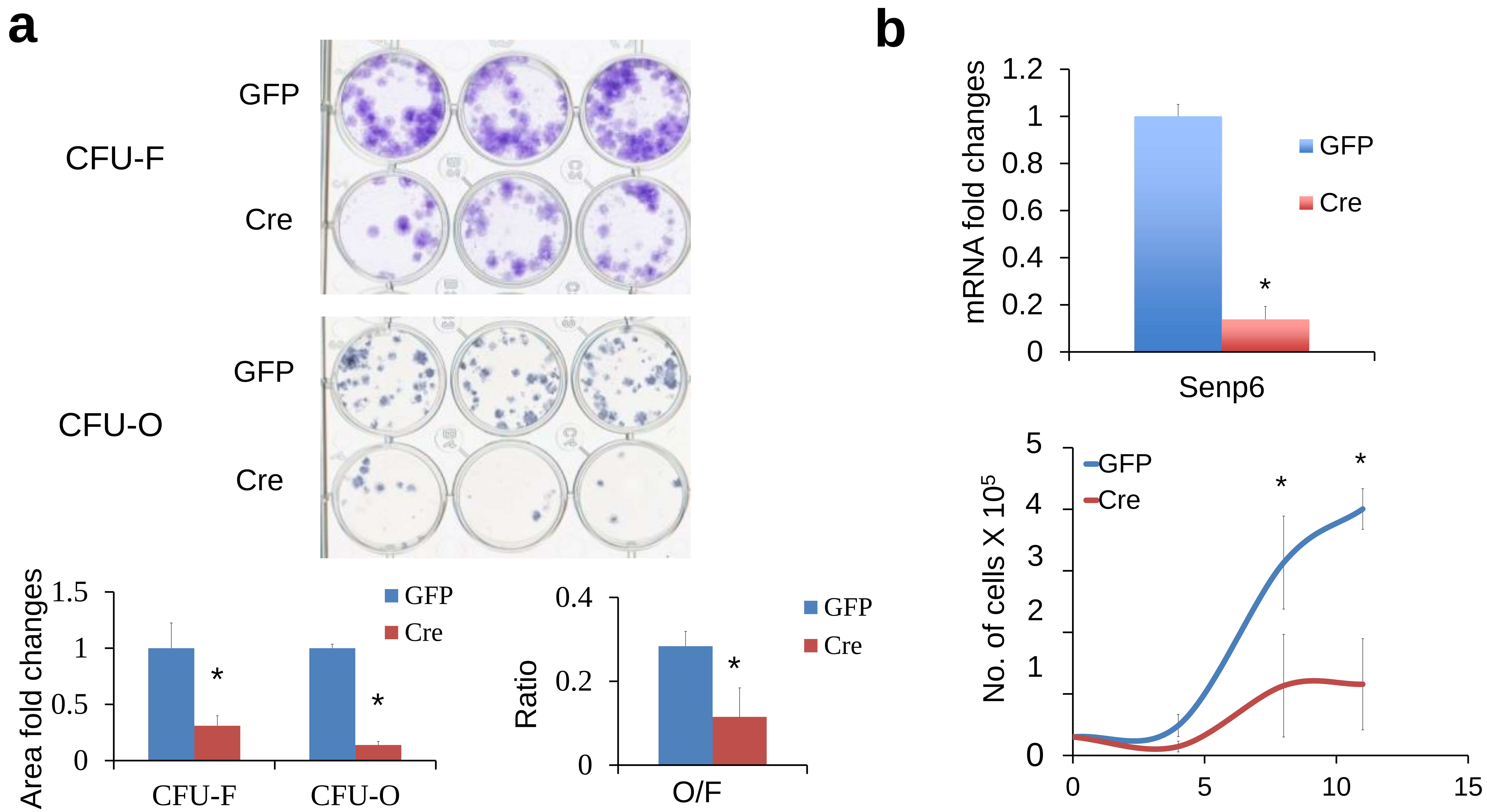

Supplementary Figure 7. *Senp6* loss caused decreased BMSC cell growth.
a. Colony-forming unit assays (CFU) show that *Senp6*$^{-/-}$ BMSCs (denoted with Cre) had decreased CFU-fibroblast (CFU-F) and CFU-osteoblast (CFU-O) (n = 3, error bars = standard deviation, **p*<0.05, compared to GFP group, Student's t-test). **b**. *Senp6*$^{-/-}$ BMSCs showed dramatically decreased cell population and reached a growth plateau after day 8 in culture. (n = 3**,** error bars = standard deviation, **p*<0.05, compared to GFP group, Student's t-test).

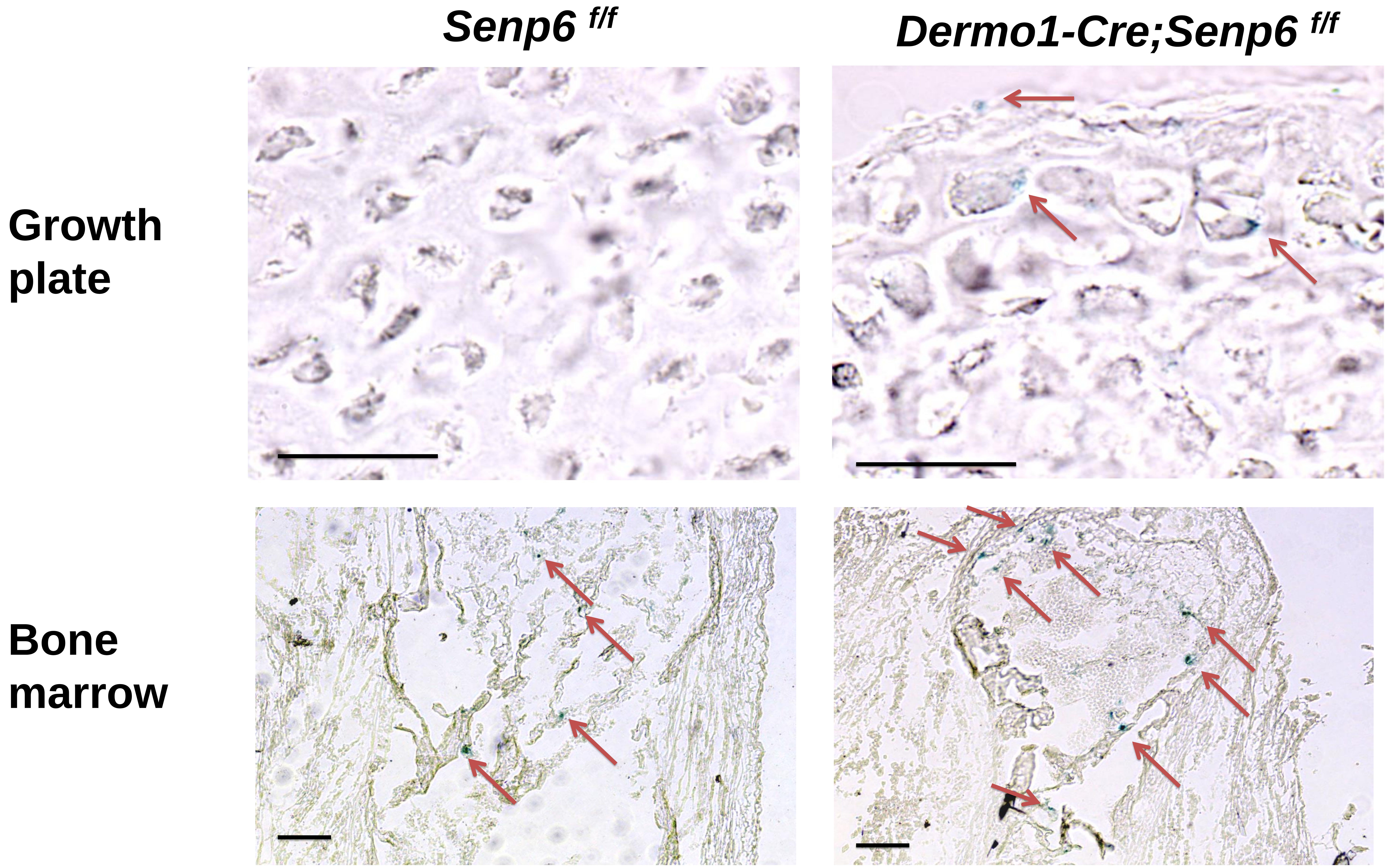

Supplementary Figure 8. Increased SA-β-Gal positive cells (indicated by red arrows) in the growth plate and bone marrow sections of the E18.5 *Dermo1-Cre;Senp6*$^{f/f}$ vs. WT mice. (scale bars = 10 μm for the growth plate and 50μm for the bone marrow).

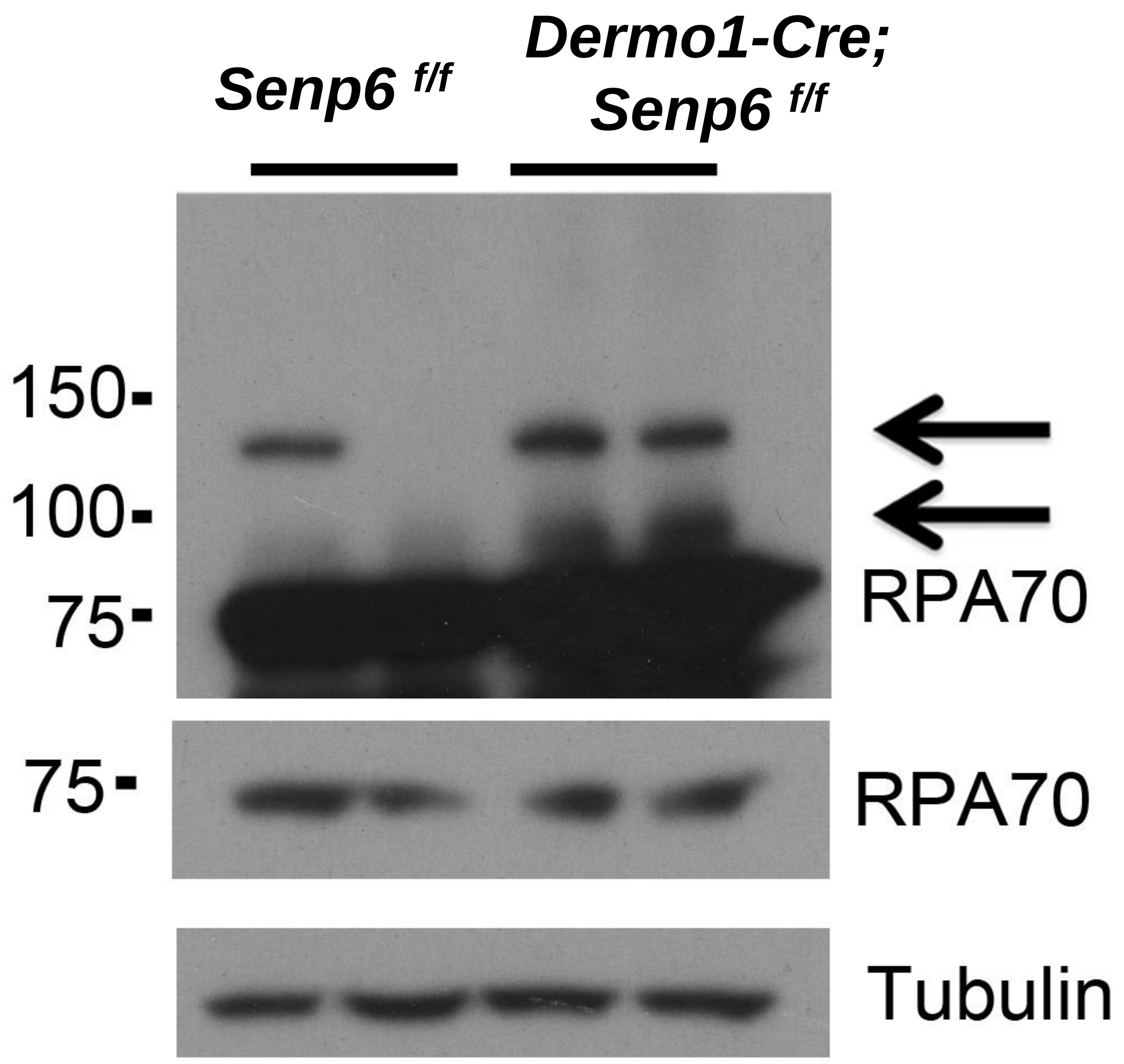

Supplementary Figure 9. *Senp6*-deficient cartilage (E18.5) showed enhanced RPA70 sumoylation. Upper panel shows the long exposure, and lower panel shows the short exposure of the same blot. The arrows indicate the sumoylated RPA70 bands. Tubulin was used as a loading control. (n = 2)

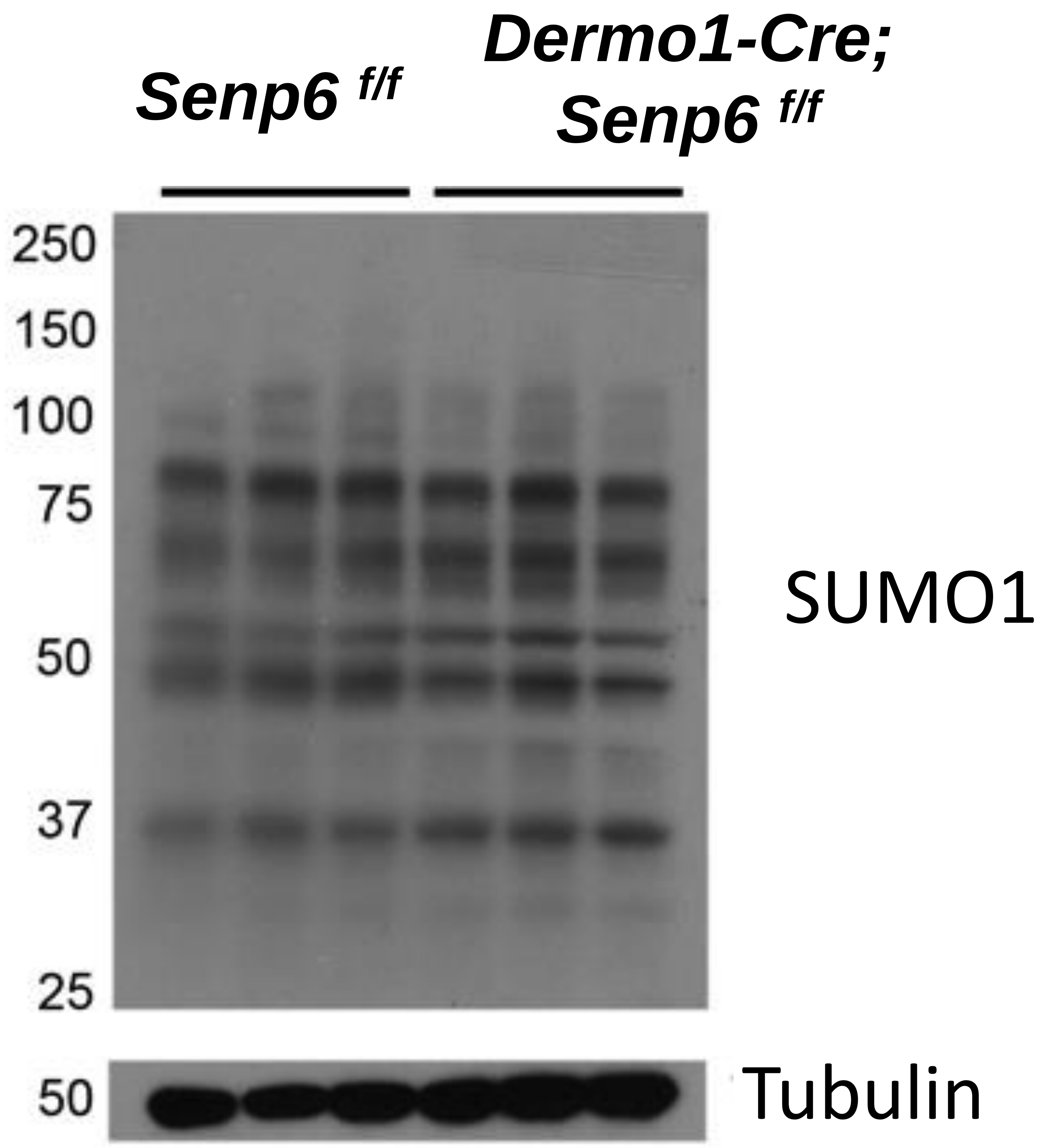

Supplementary Figure 10. *Senp6* loss in the E18.5 cartilage did not change the amount of total SUMO1 modified proteins (n = 3).

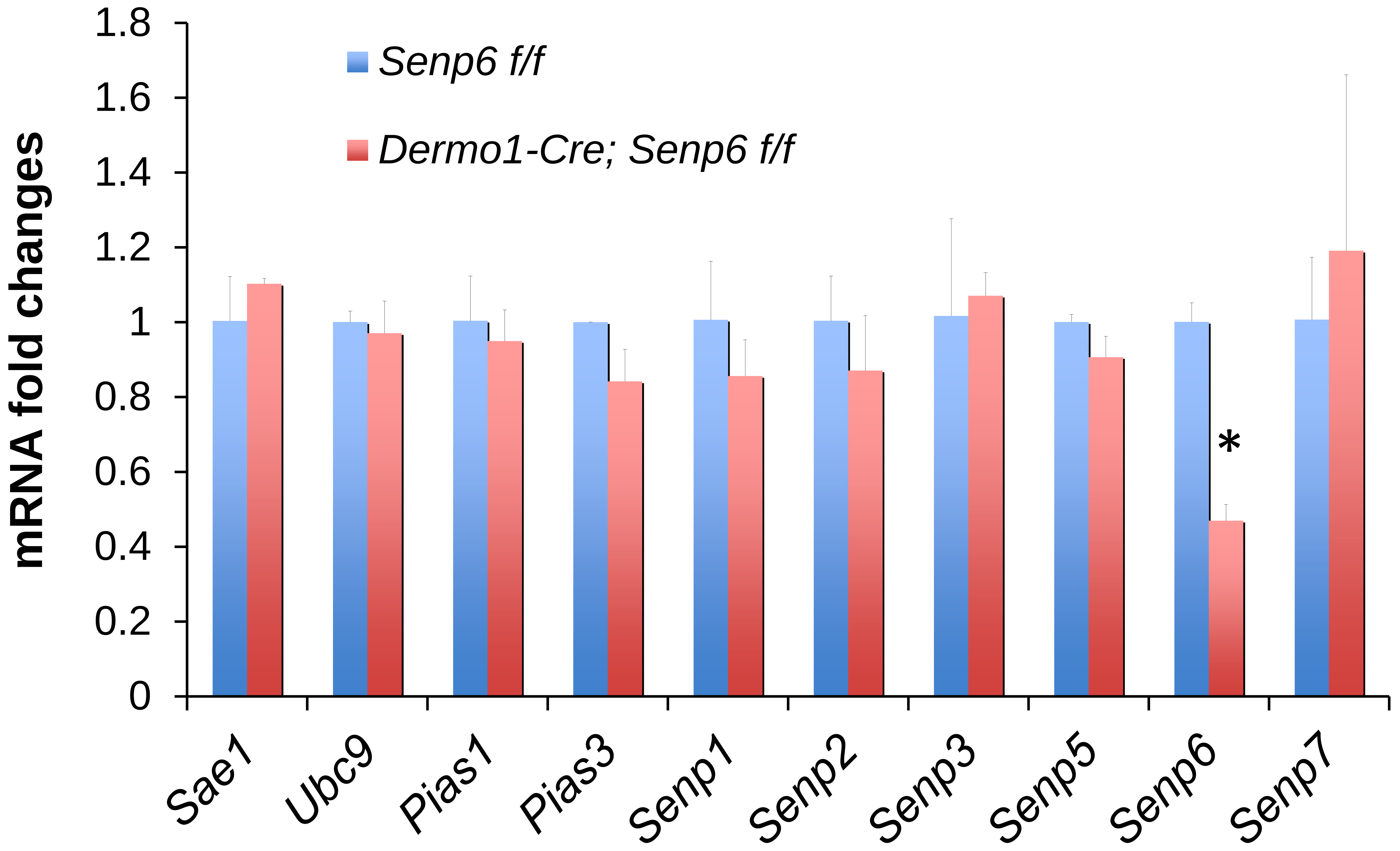

Supplementary Figure 11. *Senp6* loss did not affect the expression of other key components of sumoylation pathway in the E18.5 rib cartilages. (n = 3, error bars = standard deviation,*p < 0.05, compared with *Senp6*$^{f/f}$ controls, Student's t-test)

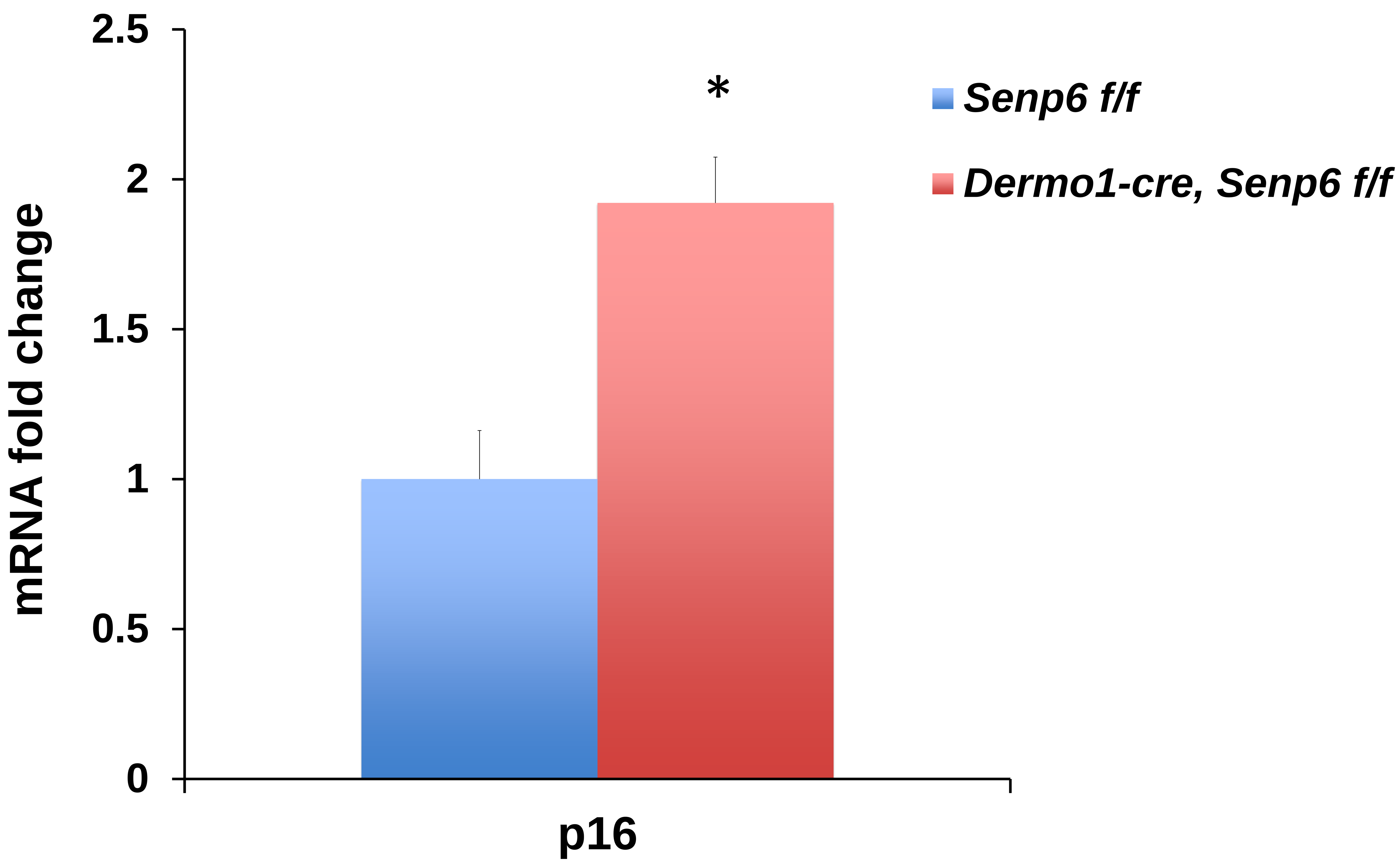

Supplementary Figure. 12. Elevated p16 expression in the E18.5 *Dermo1-Cre;Senp6*$^{f/f}$ rib cartilage (n = 3, error bars = standard deviation,*p < 0.05, compared with *Senp6*$^{f/f}$ controls, Student's t-test)

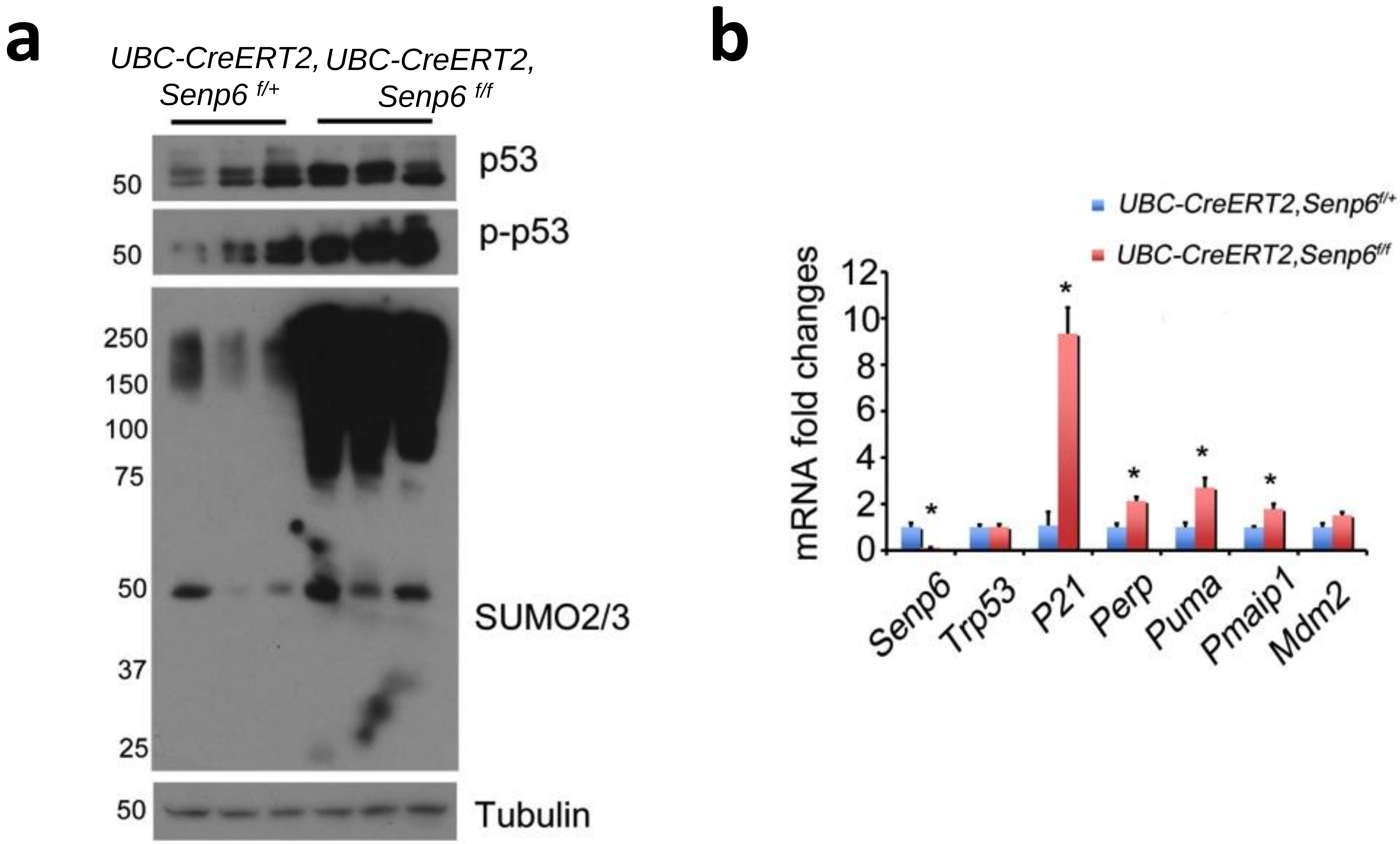

Supplementary Figure 13. *Senp6* loss activated p53 pathway in the tail of tamoxifen-treated *UBC-CreERT2;Senp6*$^{f/f}$ mice (2-mo-old mice were treated with tamoxifen once/day for 5 days; samples were harvested 2 weeks after treatments). **a**. Western blots for p53, p-p53 and SUMO2/3 modified proteins (n = 3). **b**. Expression changes of p53 downstream genes by qRT-PCR (n = 3, error bars = standard deviation,*$p < 0.05$, compared with the *UBC-CreERT2; Senp6*$^{f/+}$ controls, Student's t-test)

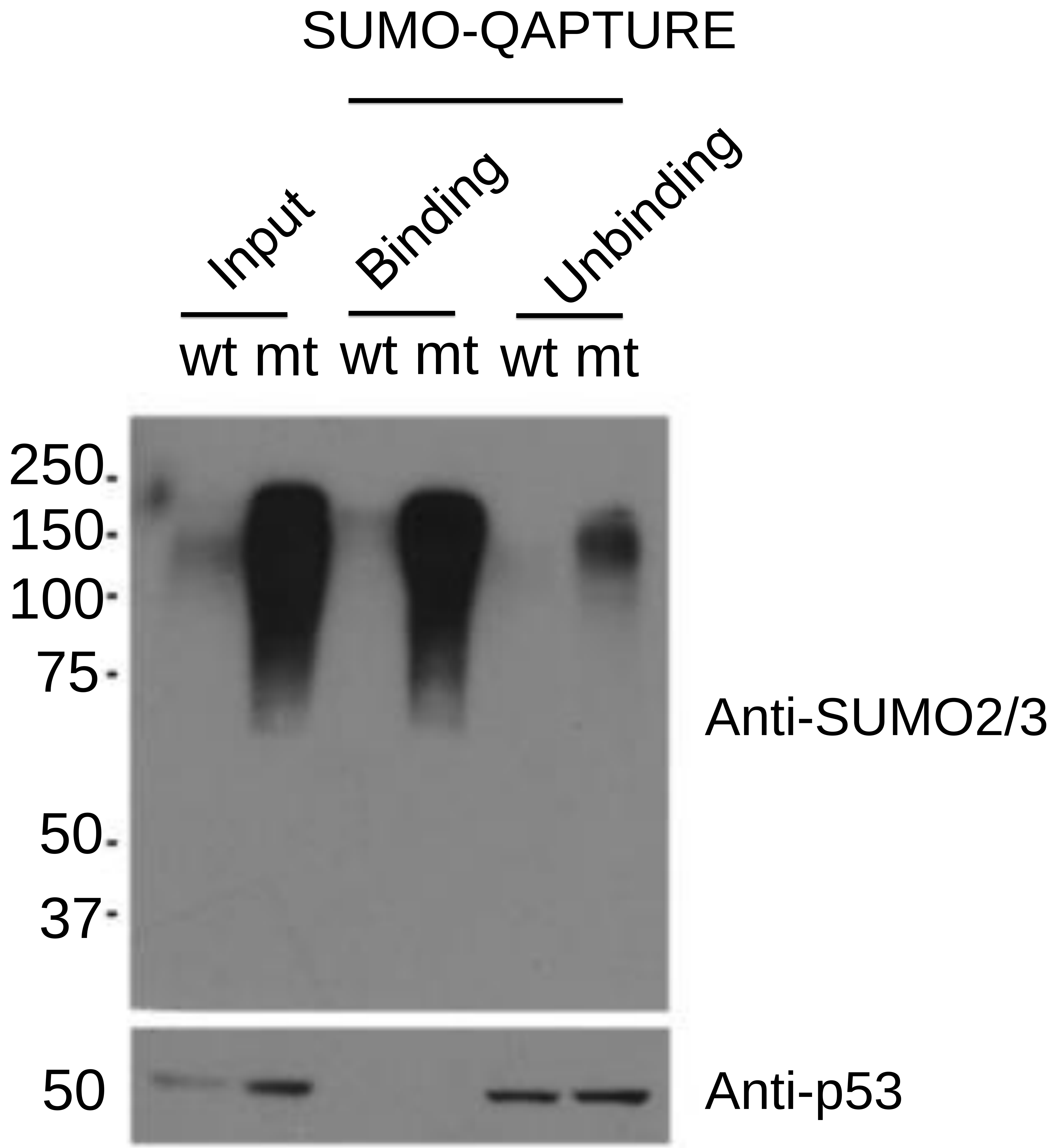

Supplementary Figure 14. The total sumoylated proteins from WT and *Senp6*$^{-/-}$ chondrocytes does not contain p53. The protein lysates of WT and *Senp6*$^{-/-}$ chondrocytes were purified by SUMO-QAPTURE affinity matrix (Enzo life science). The input, matrix-bound fraction, and unbound fraction were subjected to western blots using SUMO2/3 antibody (upper panel) and p53 antibody (lower panel) (n = 2)

Identified protein	M.W.	MT hits	WT hits	Norm. Factor	Fold changes
Junction plakoglobin OS=Mus musculus GN=Jup PE=1 SV=3	82 kDa	10	2	1.572	**7.86**
Polymerase I and transcript release factor OS=Mus musculus GN=Ptrf PE=1 SV=1	44 kDa	5	1	1.572	**7.86**
Thy-1 membrane glycoprotein OS=Mus musculus GN=Thy1 PE=1 SV=1	18 kDa	4	1	1.572	**6.29**
Protein Ahnak OS=Mus musculus GN=Ahnak PE=1 SV=1	604 kDa	14	4	1.572	**5.50**
RNA-binding protein 3 OS=Mus musculus GN=Rbm3 PE=2 SV=1	17 kDa	3	1	1.572	**4.72**
Ferritin light chain 1 OS=Mus musculus GN=Ftl1 PE=1 SV=2	21 kDa	3	1	1.572	**4.72**
tRNA-splicing ligase RtcB homolog OS=Mus musculus GN=Rtcb PE=2 SV=1	55 kDa	3	1	1.572	**4.72**
Caprin-1 OS=Mus musculus GN=Caprin1 PE=1 SV=2	78 kDa	3	1	1.572	**4.72**
Reticulon-4 OS=Mus musculus GN=Rtn4 PE=1 SV=2	127 kDa	8	4	1.572	**3.14**
Transcription intermediary factor 1-beta OS=Mus musculus GN=Trim28 PE=1 SV=3	89 kDa	6	3	1.572	**3.14**
Inactive tyrosine-protein kinase 7 OS=Mus musculus GN=Ptk7 PE=1 SV=1	118 kDa	4	2	1.572	**3.14**
RNA-binding protein EWS OS=Mus musculus GN=Ewsr1 PE=1 SV=2	68 kDa	4	2	1.572	**3.14**
Ras-related C3 botulinum toxin substrate 1 OS=Mus musculus GN=Rac1 PE=1 SV=1	21 kDa	2	1	1.572	**3.14**
LIM and SH3 domain protein 1 OS=Mus musculus GN=Lasp1 PE=1 SV=1	30 kDa	2	1	1.572	**3.14**
Isoform 2 of Septin-11 OS=Mus musculus GN=Sept11	49 kDa	2	1	1.572	**3.14**
Isoform 2 of Interleukin enhancer-binding factor 3 OS=Mus musculus GN=Ilf3	78 kDa	2	1	1.572	**3.14**
Spectrin beta chain, non-erythrocytic 1 OS=Mus musculus GN=Sptbn1 PE=1 SV=2	274 kDa	8	5	1.572	**2.52**
Isoform 2 of Fibulin-2 OS=Mus musculus GN=Fbln2	126 kDa	9	6	1.572	**2.36**
Vinculin OS=Mus musculus GN=Vcl PE=1 SV=4	117 kDa	6	4	1.572	**2.36**
S-formylglutathione hydrolase OS=Mus musculus GN=Esd PE=1 SV=1	31 kDa	6	4	1.572	**2.36**
Cluster of 26S proteasome non-ATPase regulatory subunit 2 OS=Mus musculus GN=Psmd2 PE=1 SV=1 (sp|Q8VDM4|PSMD2_MOUSE)	100 kDa	3	2	1.572	**2.36**
Cluster of Small ubiquitin-related modifier 2 OS=Mus musculus GN=Sumo2 PE=1 SV=1 (sp|P61957|SUMO2_MOUSE)	11 kDa	3	2	1.572	**2.36**
Asparagine--tRNA ligase, cytoplasmic OS=Mus musculus GN=Nars PE=1 SV=2	64 kDa	3	2	1.572	**2.36**
Catenin beta-1 OS=Mus musculus GN=Ctnnb1 PE=1 SV=1	85 kDa	3	2	1.572	**2.36**
Isoform 2 of Filamin-C OS=Mus musculus GN=Flnc	287 kDa	16	11	1.572	**2.29**
Protein Cald1 OS=Mus musculus GN=Cald1 PE=1 SV=1	62 kDa	14	10	1.572	**2.20**
Heterogeneous nuclear ribonucleoprotein A/B OS=Mus musculus GN=Hnrnpab PE=1 SV=1	31 kDa	7	5	1.572	**2.20**

Supplementary Figure 15. List of the SUMO-modified proteins that were elevated in the *Dermo1-Cre;Senp6*$^{f/f}$ rib chondrocytes.

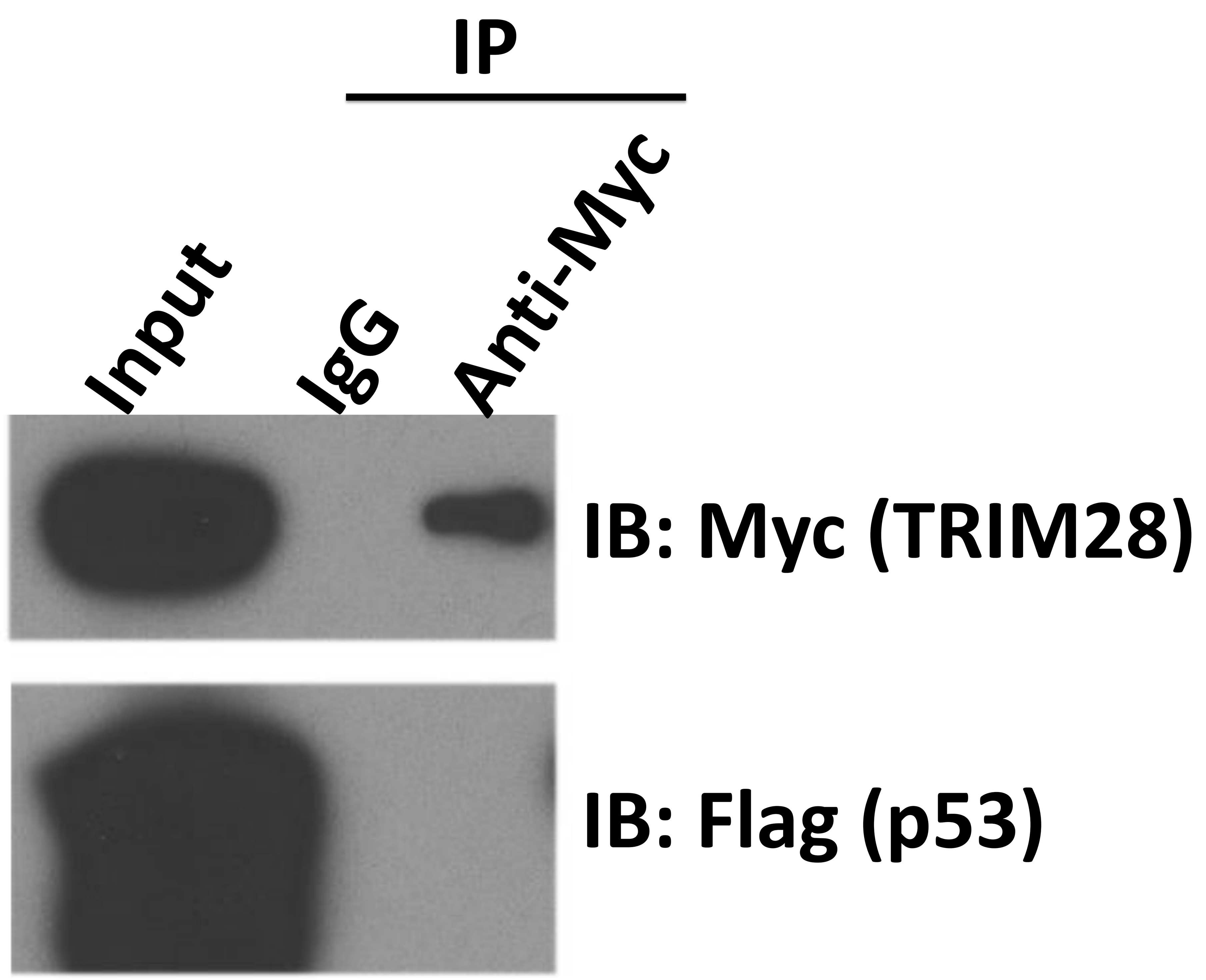

Supplementary Figure 16. TRIM28 did not co-immunoprecipitate with p53

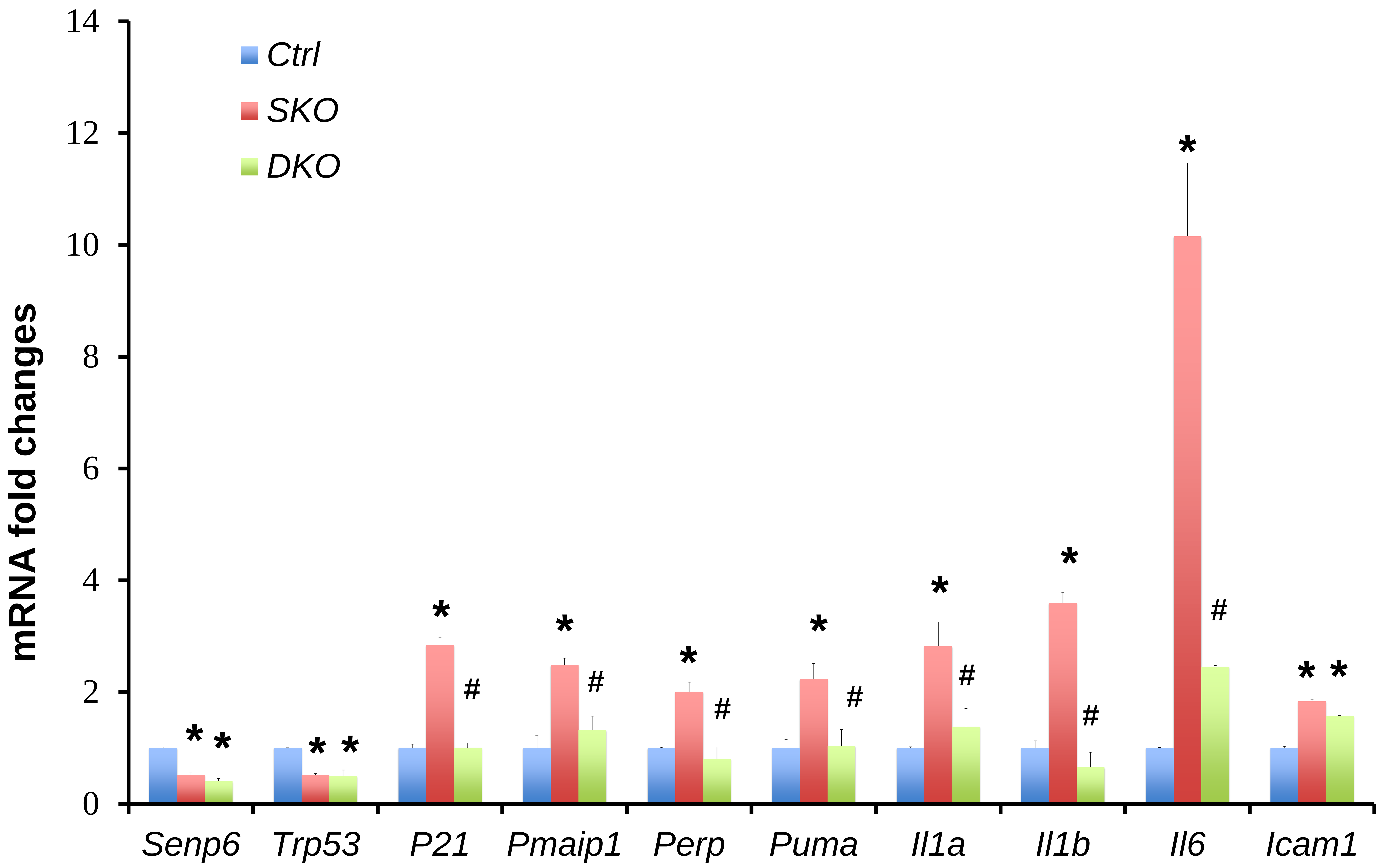

Supplementary Figure 17. The calvarias samples from control (Ctrl), SKO, and DKO mice show p53 downstream targets and SASP markers, recapitulating those detected in rib cartilage (see **Fig. 4e)** (Error bars = standard deviation, *p < 0.05, n = 3, compared to Ctrl. $^{\#}$p < 0.05, n = 3, compared to SKO, Student's t-test).

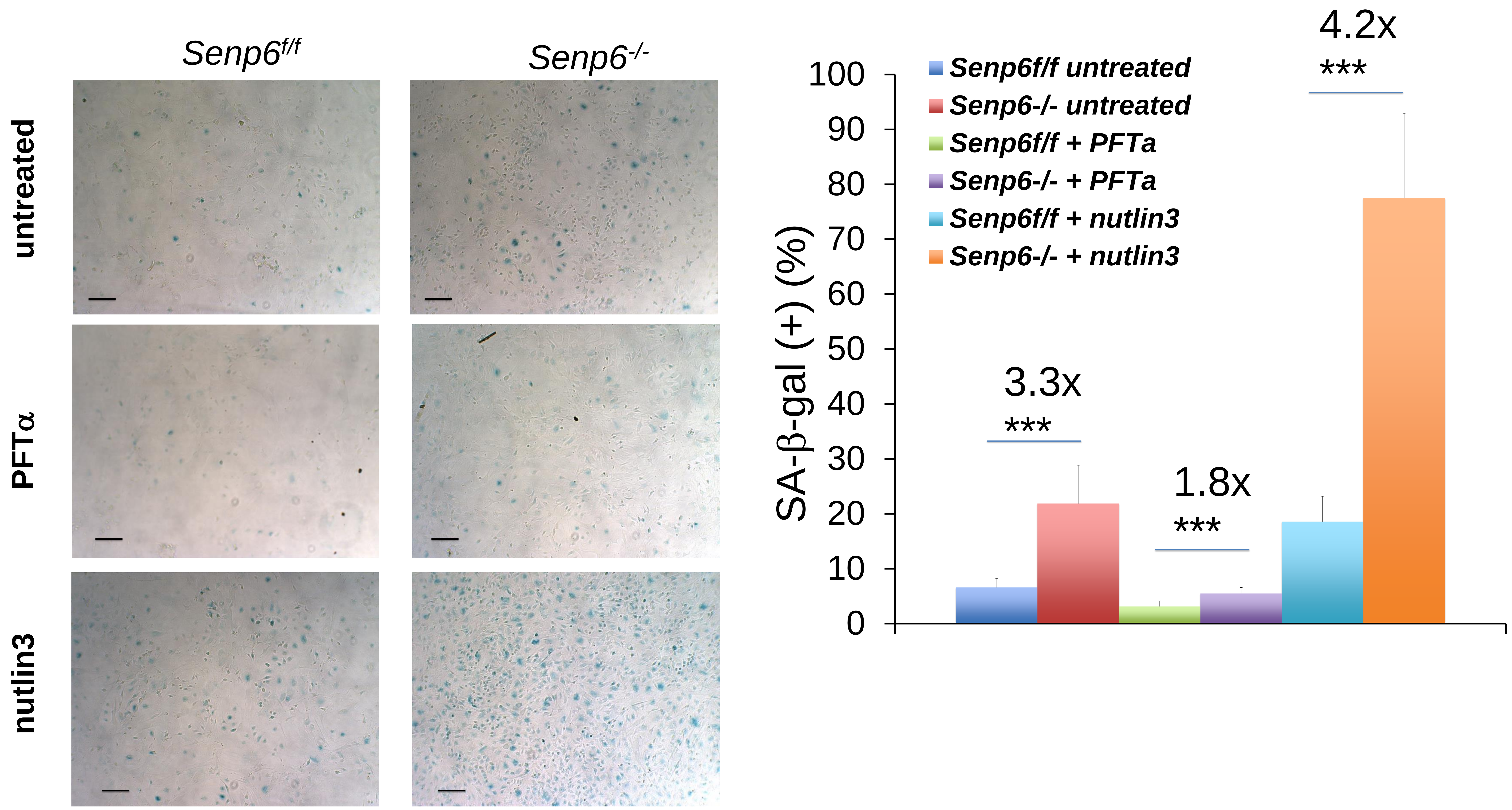

Supplementary Figure 18. *Senp6* loss-induced chondrocyte senescence was corrected by p53 inhibitor PFTα and was augmented by p53 agonist nutlin3. Quantification is shown in the graph at right. (n = 3, error bars = standard deviation, *** $p < 0.0001$, Student's t-test).

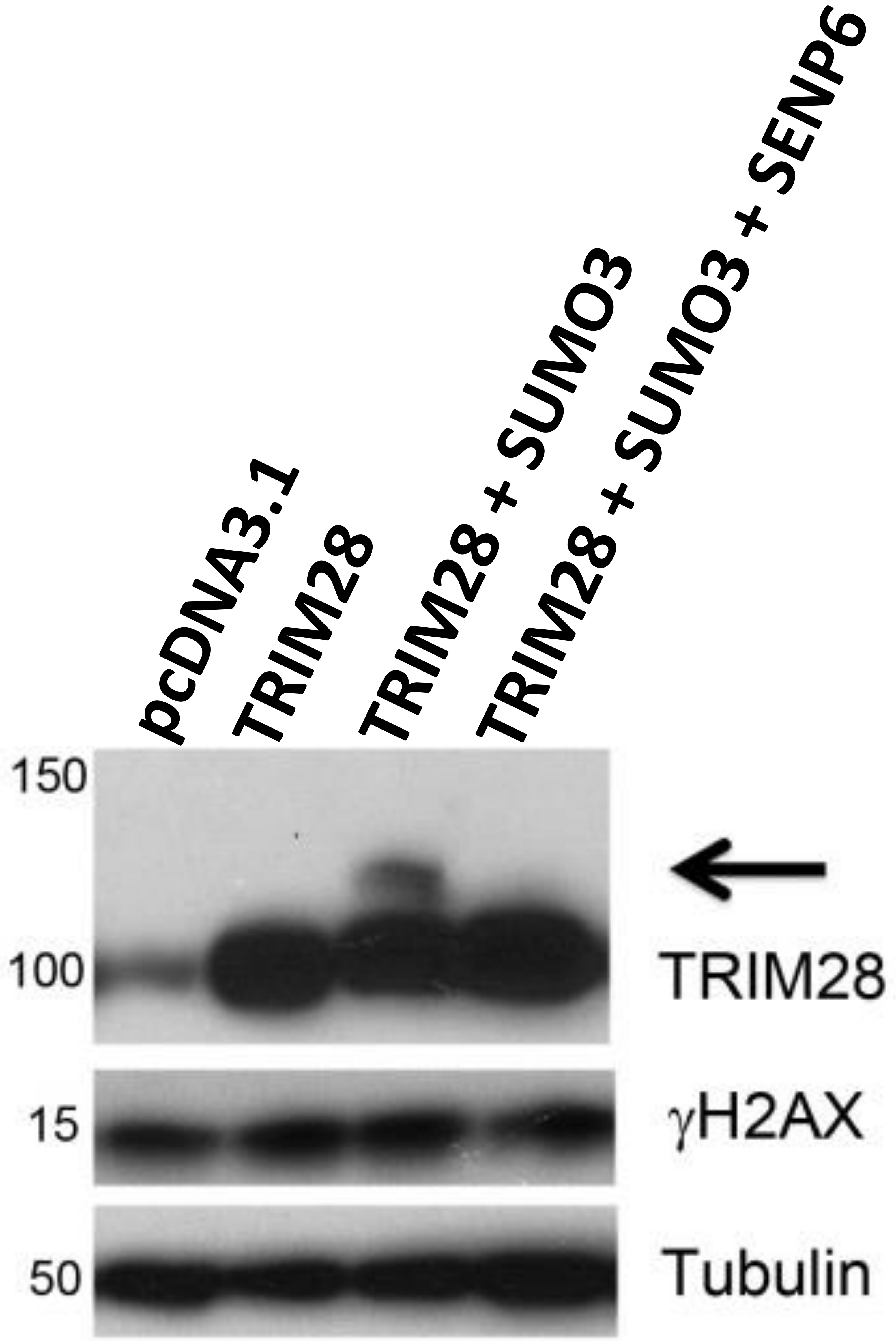

Supplementary Figure 19. Altered sumoylation of the overexpressed TRIM28 did not affect the γH2AX levels. HEK293 cells were transfected with the plasmids listed above each lane, cultured for 48 h, then subjected to western blot detections. The arrow indicates sumoylated TRIM28. Tubulin was used as a loading control.

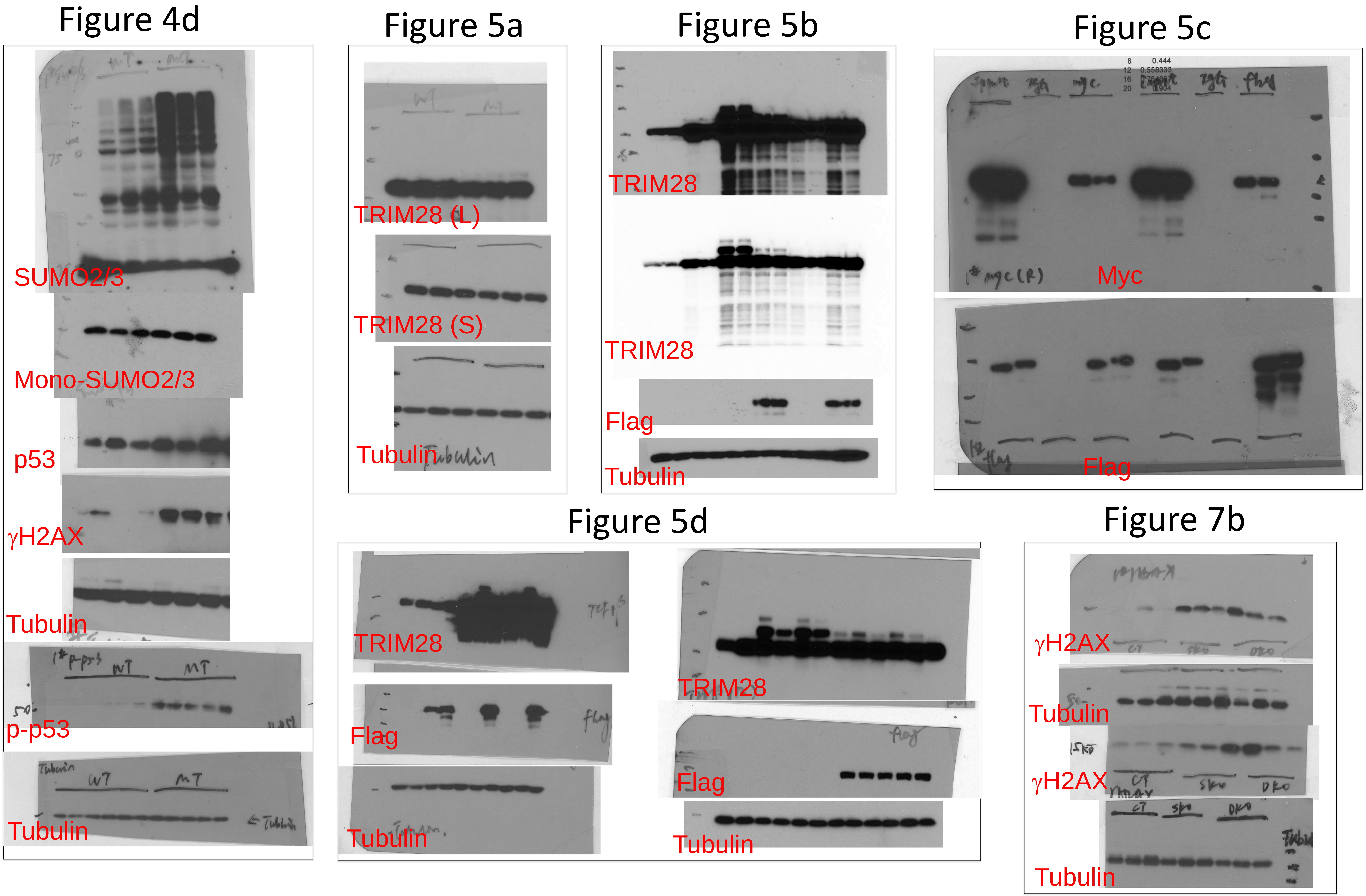

Supplementary Figure 20. Original uncropped scans of Western blots for Figure 4d, 5a-d, and 7b.

Supplementary Figure 9

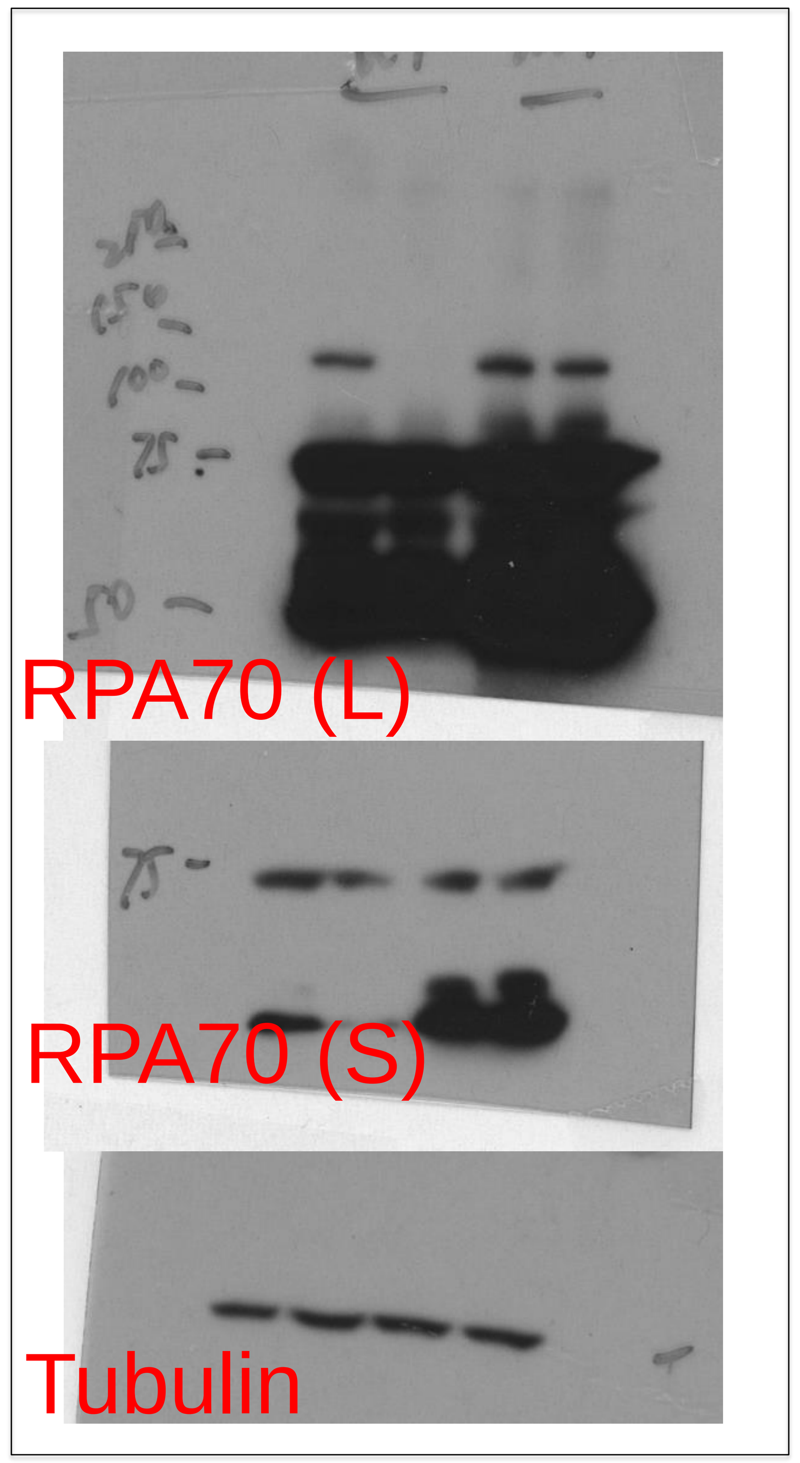

Supplementary Figure 10

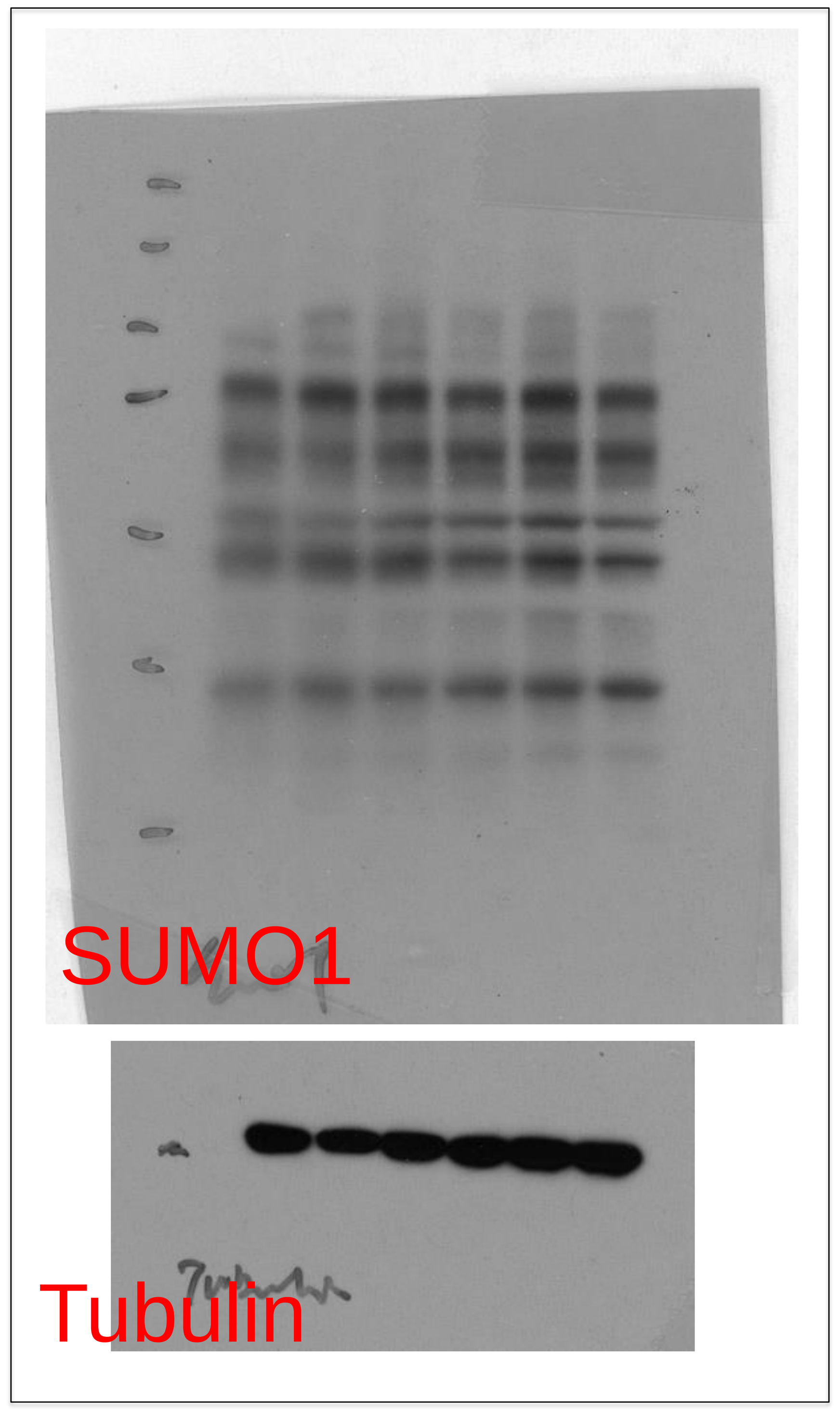

Supplementary Figure 13

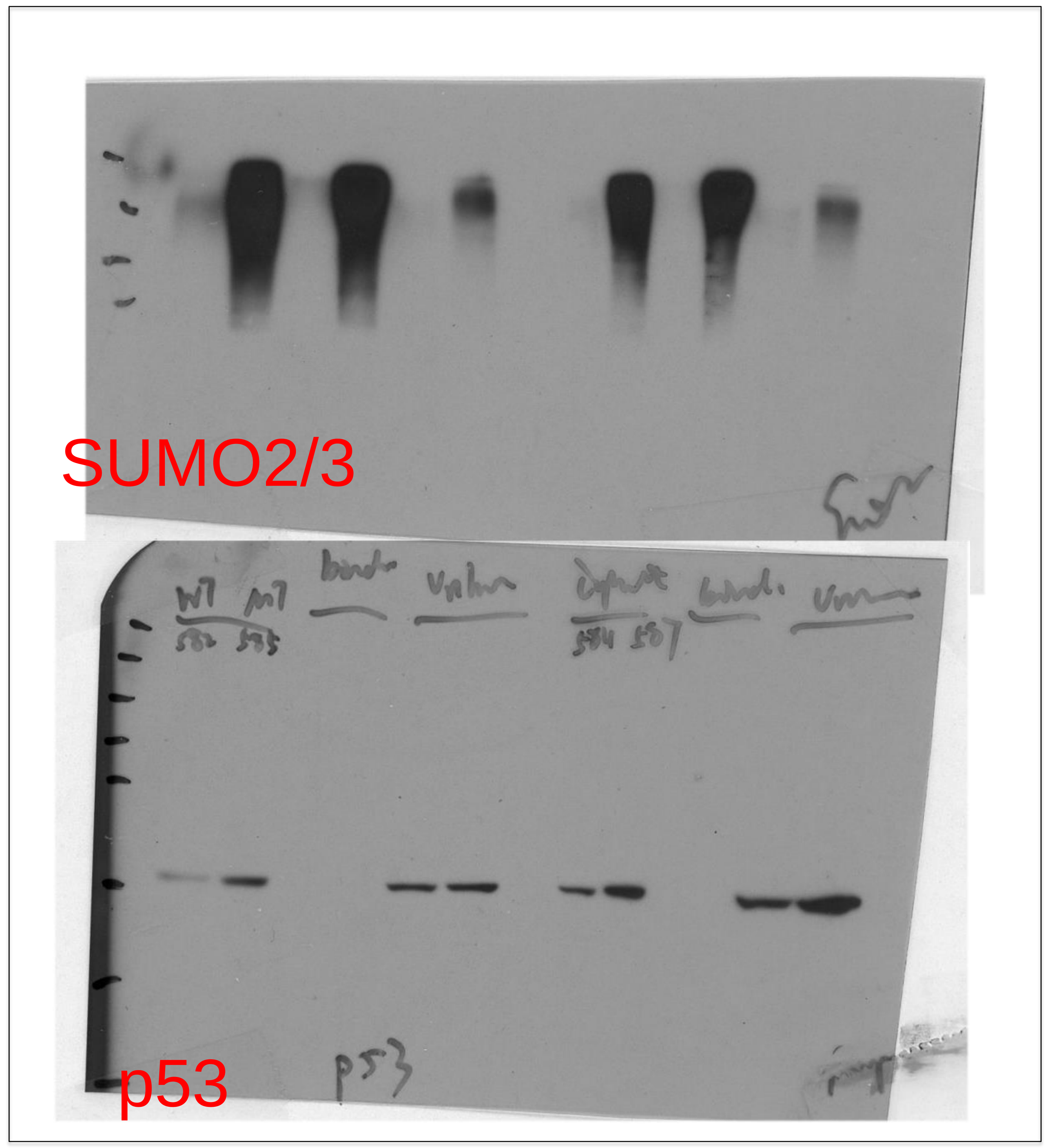

Supplementary Figure 14

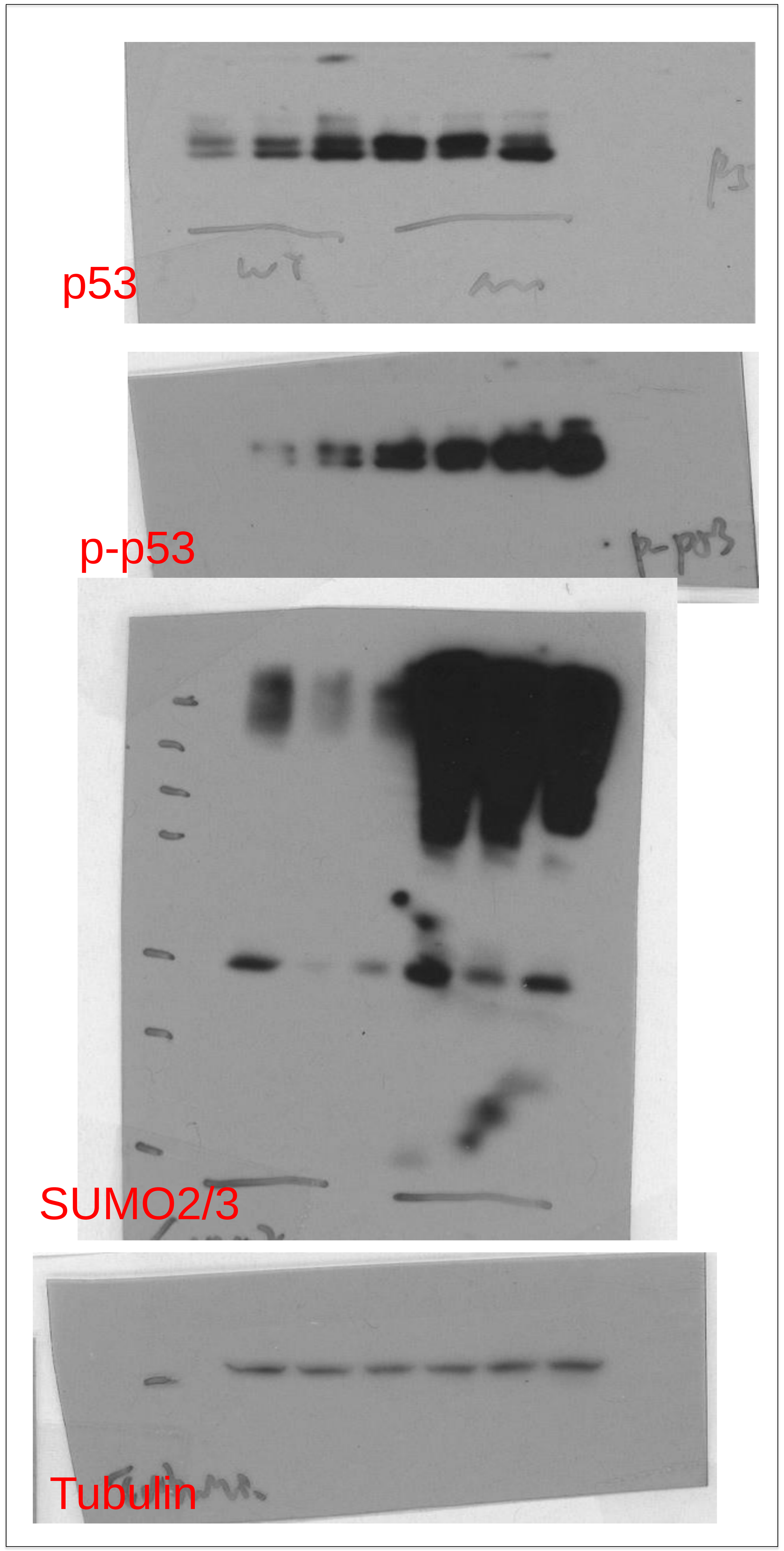

Supplementary Figure 16

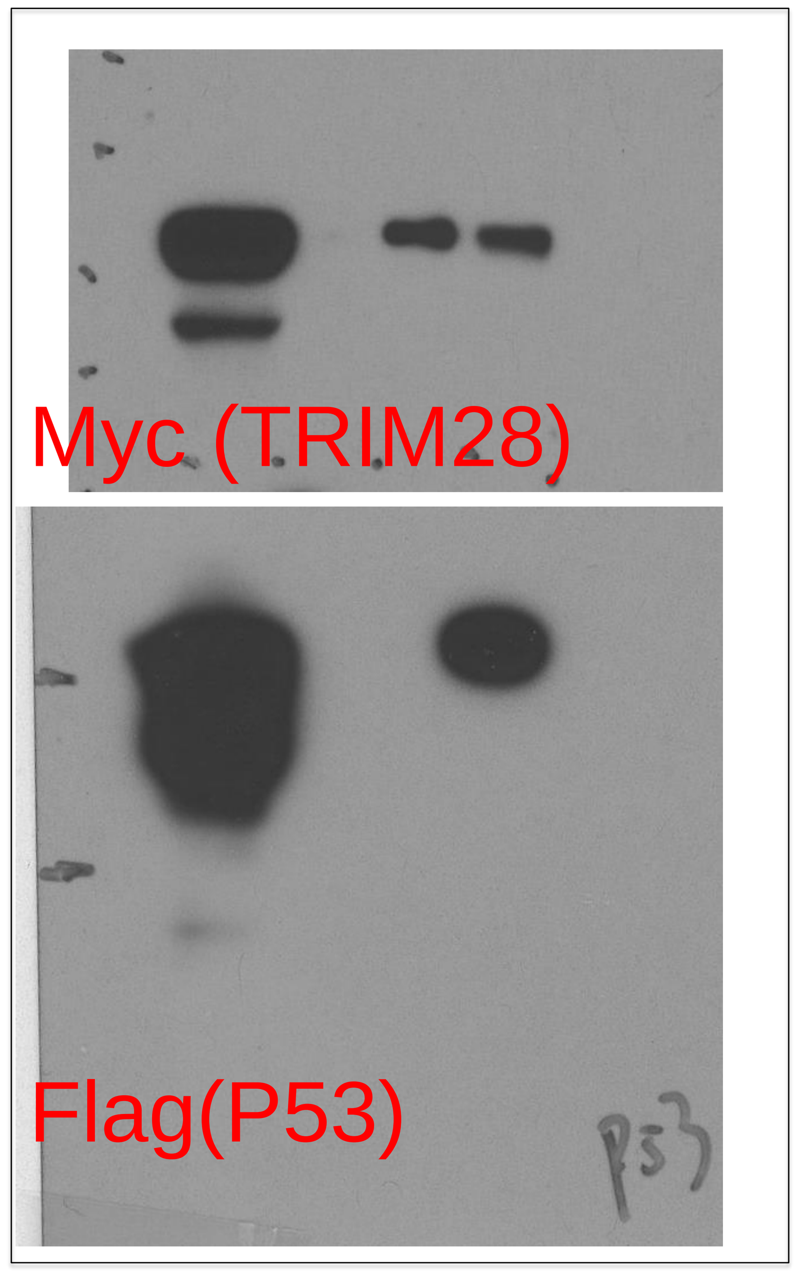

Supplementary Figure 19

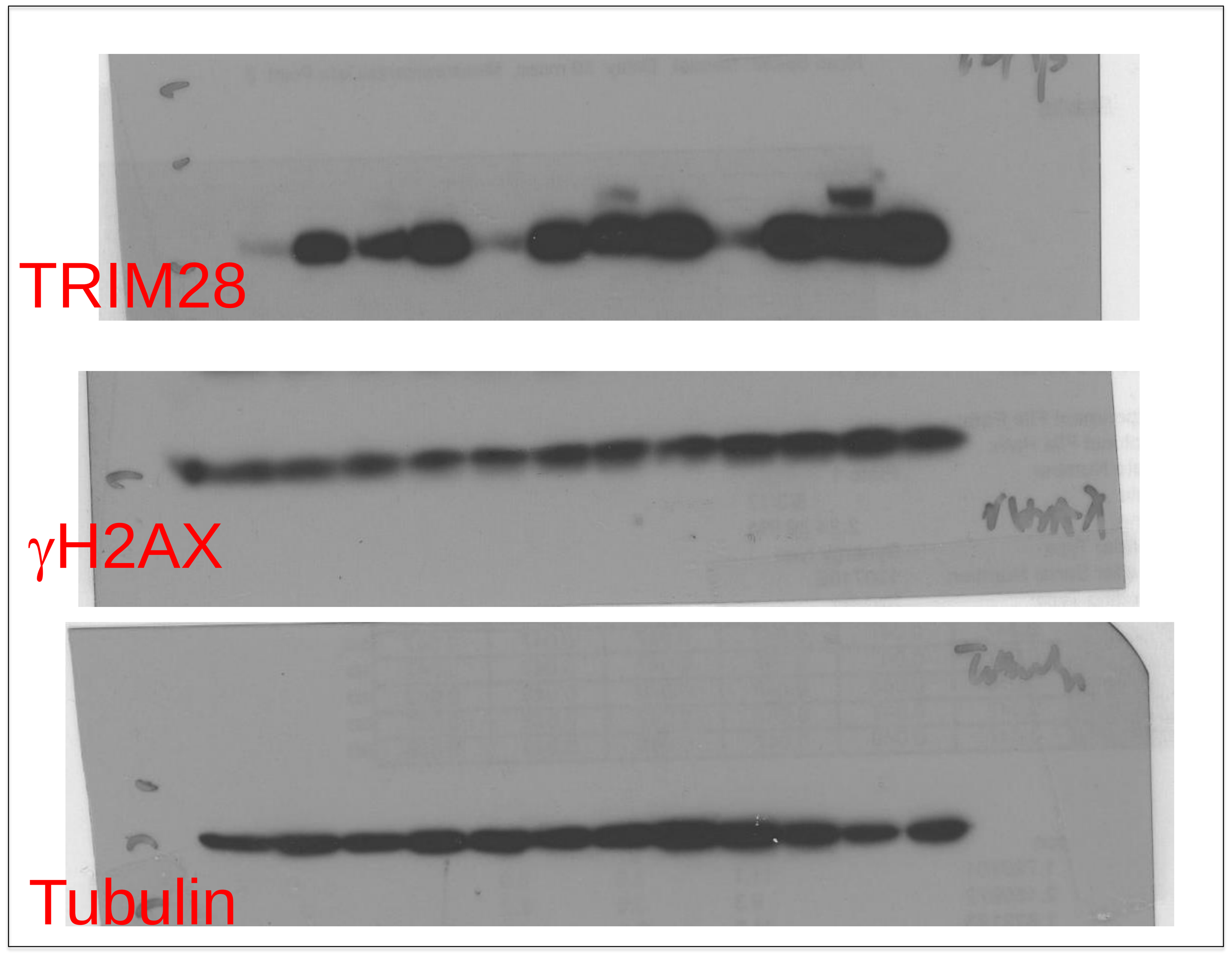

Supplementary Figure 21. Original uncropped scans of Western blots for Supplementary Figure 9, 10, 13, 14, 16, and 19.
